# Untangling Local Pro-Inflammatory, Reparative, and Regulatory Damage-Associated Molecular-Patterns (DAMPs) Pathways to Improve Transplant Outcomes

**DOI:** 10.3389/fimmu.2021.611910

**Published:** 2021-02-23

**Authors:** Gaelen K. Dwyer, Hēth R. Turnquist

**Affiliations:** ^1^ Departments of Surgery and Immunology, University of Pittsburgh School of Medicine, Pittsburgh, PA, United States; ^2^ Thomas E. Starzl Transplantation Institute, University of Pittsburgh School of Medicine, Pittsburgh, PA, United States; ^3^ McGowan Institute for Regenerative Medicine, University of Pittsburgh, Pittsburgh, PA, United States

**Keywords:** transplantation, ischemia–reperfusion injury, damage-associated molecular patterns, tissue repair and fibrosis, regulatory T cell, macrophage, alarmins, immunometabolism

## Abstract

Detrimental inflammatory responses after solid organ transplantation are initiated when immune cells sense pathogen-associated molecular patterns (PAMPs) and certain damage-associated molecular patterns (DAMPs) released or exposed during transplant-associated processes, such as ischemia/reperfusion injury (IRI), surgical trauma, and recipient conditioning. These inflammatory responses initiate and propagate anti-alloantigen (AlloAg) responses and targeting DAMPs and PAMPs, or the signaling cascades they activate, reduce alloimmunity, and contribute to improved outcomes after allogeneic solid organ transplantation in experimental studies. However, DAMPs have also been implicated in initiating essential anti-inflammatory and reparative functions of specific immune cells, particularly Treg and macrophages. Interestingly, DAMP signaling is also involved in local and systemic homeostasis. Herein, we describe the emerging literature defining how poor outcomes after transplantation may result, not from just an over-abundance of DAMP-driven inflammation, but instead an inadequate presence of a subset of DAMPs or related molecules needed to repair tissue successfully or re-establish tissue homeostasis. Adverse outcomes may also arise when these homeostatic or reparative signals become dysregulated or hijacked by alloreactive immune cells in transplant niches. A complete understanding of the critical pathways controlling tissue repair and homeostasis, and how alloimmune responses or transplant-related processes disrupt these will lead to new immunotherapeutics that can prevent or reverse the tissue pathology leading to lost grafts due to chronic rejection.

## Introduction

Tissue injury negatively impacts outcomes after the transplantation (Tx) of cells, tissues, or organs. In solid organ transplantation (SOTx) and vascularized composite allograft (VCA) transplantation ischemia reperfusion injury (IRI), surgical manipulations, donor trauma, and brain death initiate the release of self-derived molecules containing damage-associated molecular patterns (DAMPs) that alert immune cells to the damage (1, 2). Released DAMPs will act on resident donor and graft-infiltrating immune cells to shape local and systemic immune functions that determine SOTx short and long-term outcomes. Some DAMPs are not released from necrotic cells, but instead are exposed on stressed or dying cell membranes (3). This review briefly discusses recent advances in understanding how DAMPs contribute to inflammation that stimulates alloimmunity, but highly detailed information can be found in numerous excellent reviews (1, 2, 4–6). In this review, we also elaborate on emerging concepts in Tx that are developing from an evolving understanding of the potential beneficial function of DAMPs in tissue repair and systemic homeostasis. We will also discuss examples of how these reparative or homeostatic DAMP pathways may become dysregulated or re-appropriated throughout the graft by anti-donor immune responses to also contribute to chronic rejection (CR).

## Current Understanding of DAMPs as Drivers of Alloimmune Responses and Poor Outcomes After SOTx

Numerous DAMPs released during cellular stress, tissue injury, or *via* inflammatory cell death pathways such as ferroptosis, necroptosis, pyroptosis, have been identified (6, 7). How these DAMPs initiate sterile inflammation and contribute to anti-AlloAg immune responses has been reviewed recently (1, 2, 4, 5). Well-characterized pro-inflammatory DAMPs active in Tx include nuclear materials, such as high-mobility group box 1 (HMGB1), interleukin (IL)-1α, cytoplasmic components, including ATP, heat shock proteins (HSPs), and s100 proteins, mitochondrial (mt) contents like mtDNA or mt transcriptional factor A, as well as extracellular matrix (ECM) components, including hyaluronan, fibronectin, and heparan sulfate have been assessed in Tx models (**Table 1**). Oxidative injury-induced neo-antigens and typically sequestered cytoplasmic proteins such as HSPs and the ER chaperone calreticulin can be exposed on or incorporated in the cell membrane.

**Table 1:** Inflammatory DAMPs and their impact on SOTx outcomes.

Family	Molecule	Receptors	Role in Tx- related inflammation/immunity/outcomes	References
Inflammatory DAMPs				
	Histones	TLR2, TLR4, and TLR9	Causes TLR- and inflammasome-dependent generation of inflammatory response by innate cells	(6, 8, 9)
	HMGB1	TLR2, TLR4, RAGE, and TIM3	Promotes the production of pro-inflammatory cytokines and chemokines by innate immune cells Induces metabolic reprogramming supporting the pro-inflammatory functions of myeloid APC Promotion of AR and CR in experimental models Implicated in poor outcomes after clinical transplantation	(2, 10–15)
	IL1α	IL-1R	Promotes the production of pro-inflammatory cytokines and chemokines by innate immune cells	(16)
	ATP	P2Y2 and P2X7	Attraction and activation of innate cells Promotes inflammasome activity Causes the release pro-inflammatory cytokines supporting rejection Promotes IL-1β and IL18 secretion and initiates inflammatory cell death Stimulates alloimmunity	(6, 17–20)
	Vimentin	Dectin-1	Induces metabolic reprogramming supporting the pro-inflammatory functions of myeloid APC Induces macrophage TNF α and IL-6 production	(6, 10)
	Hyaluronan (HA)	TLR2 and TLR4	Low molecular weight breakdown products stimulate macrophages pro-inflammatory cytokine production Supports of alloimmunity	(21–23)
	S100s	TLR2, TLR4, RAGE	Potent immunostimulatory activity, monocytes and neutrophils recruitment	(6, 24)
	Mitochondrial DNA (mDNA)	TLR9	Macrophages and neutrophils activation Promotes inflammasome activity Causes the release pro-inflammatory cytokines supporting rejection Promotes IL-1β and IL18 secretion and initiates inflammatory cell death	(6, 25, 26)

Many defined DAMPs are recognized by conserved pattern recognition receptors (PRRs) that also recognize non-self materials containing pathogen-associated molecular patterns (PAMPs) to generate protective immune responses (**Table 1**). This overlap has made it difficult to clearly define the role of specific DAMPs versus bacterial product contamination in early studies (6). Nevertheless, studies with antibodies targeting specific DAMPs, as well as the generation and use of DAMP KO mice, have established that self-derived materials influence SOTx outcomes in experimental transplant models (5). Other PRR-type receptors able to detect DAMPs include NOD-like receptors (NLRs), retinoic acid-inducible gene I (RIG-I)-like receptor (RLRs), C-type lectin receptors, and intracellular DNA sensors, such as cyclic GMP-AMP synthase (cGAS), and absent in melanoma 2 (AIM2) (6). Non-PRR receptors such as the receptor for advanced glycation end products (RAGE) as well as G-protein-coupled receptors like formyl peptide receptors (FPRs) and P2Y receptors, detecting extracellular nucleotides like ATP have been revealed to be important in sterile inflammation and supporting alloimmune responses leading to acute rejection (AR) and CR (1, 2). Exposed HSPs and calreticulin displayed on the cell surface can be recognized by CD91 and aid engulfment and presentation of alloAg by antigen presenting cells APC (27, 28). Natural antibody responses to displayed oxidative-induced neo-antigens can also trigger APC activation *via* the complement cascade (3, 4). DAMPs stimulation of pro-inflammatory immune response leading to increased alloimmunity is well appreciated to contribute significantly to both AR and CR in experimental models (10–12) and this important subject has been the focus of several recent and thorough reviews (1, 2, 4, 5). Clinically, DAMPs are implicated in AR of liver grafts (13) as well as CR of cardiac transplants (14). HMGB1 is induced by IRI in cadaveric kidney transplants, but absent from living donor grafts that have better outcomes (15). Similarly, recipients with a mutation in TLR4 that decreases the affinity for HMGB1 exhibit better early graft function (15).

The concept that the presence of DAMPs leads to poor early and late Tx outcomes is supported by the clinical observation that shorter IRI times result in reduced risk of AR and CR after SOTx. The finding that HLA mismatch, recipients of living, unrelated donor kidneys have significantly better long-term outcomes relative to those receiving HLA-matched cadaveric kidney grafts subjected to longer periods of ischemia support this premise (29). Furthermore, each hour of cold ischemia increases the odds of AR (30), early graft failure, and mortality after kidney Tx (31). Similar negative associations with outcomes with increased ischemia times have been made for cardiac (32, 33) and liver Tx (34, 35).

## Directly Targeting DAMP Signaling to Improve Outcomes

Clinical observations supporting DAMPs as a dominant initiator of IRI and contributor to alloimmunity and rejection have compelled efforts to antagonize them after SOTx. As outlined in **Table 1**, many DAMPs initiate function by activating TLRs and the downstream adapter MyD88. Pre-clinical studies using MyD88 or TLR deficient mice have identified both as effective targets to limit IRI, inflammation, and improve transplant outcomes (36). These early rodent studies utilizing TLR and MyD88 deficient mice have led to the development of numerous biologics targeting TLRs and MyD88, which have shown promise in promoting tolerance or limiting rejection (37–42). For example, newer agents like Eritoran, which is a synthetic analog of the lipid A portion of lipopolysaccharide (LPS) that can antagonize LPS binding (43, 44), or the 2-aminothiazole-derived MyD88 inhibitor TJ-M2010-5, are potent inhibitors of DC activation and promoted long-term heart and skin graft survival in rodents (37).

Nevertheless, successful translation of agents targeting these pathways remains to be realized. NI-0101 is a humanized, anti-TLR4 antibody that interferes with TLR4 dimerization and provides sustained blocking of LPS‐induced cytokine production in healthy volunteers (45, 46). It, however, failed recently to alter disease in a Phase II, randomized, placebo-controlled, double-blind, international, multicenter study of individuals with moderate to severe rheumatoid arthritis (47). OPN-305 (Tomaralimab, Opsona Therapeutics) is a humanized, IgG4 monoclonal antibody against TLR2 under recent phase II investigation to prevent delayed graft function after kidney Tx (48). Both OPN-305 and OPN-201, its murine monoclonal parent antibody, have shown a potent ability to antagonize TLR2 signaling that is activated by HMGB1 and several HSPs, and limit IRI in rodents and swine (49, 50). An earlier Phase I study established that OPN-305 infusions were well tolerated and consistently inhibited heat-killed *listeria monocytogenes*-mediated IL-6 secretion by patients’ peripheral blood cells for periods up to 90 days (51). While these studies would suggest promise, the future of OPN-305 is unclear. There have not been any published reports from the Phase II trial, and as of 2019, Opsona was liquidated after the search for a development partner or buyer for its leading drug therapy was fruitless (52). Given the importance of TLRs and MyD88 to the initiation of detrimental pro-inflammatory response to after IRI and demonstrated in pre-clinical Tx studies, it can be expected that efforts to identify clinical candidates that effectively target this pathway and limit early inflammation after Tx will persist. As trials of these agents move forward, it will be very interesting to observe if the benefits that potent TLR signaling pathway inhibitors have against DAMP-driven IRI and tissue damage and the generation of alloimmune responses can outweigh the expected blunting of effective anti-pathogen immunity (53). If this class of drugs only produces the generalized immunosuppression similar to that observed with non-specific TCR signaling inhibition with drugs like Tacrolimus, their impact will be limited. However, if potent drugs blocking TLR signaling can be delivered for only for a short window in or around Tx surgery for highly effective prevention of the general innate inflammation initiating AlloAg-specific T cell responses DAMP-activated antigen presenting cells (APC), this class of drugs blocking tissue damage-mediated inflammation could be transformative.

## Targeting Immunometabolism to Limit the Pro-Inflammatory Activity of DAMPs

Ischemia resulting from organ procurement not only causes cellular stress and cell death that releases DAMPs, but it will also cause graft hypoxia that will program graft-resident, donor immune cells and infiltrating recipient immune cells for an inflammatory response to these DAMPs. Myeloid cells in a hypoxic environment upregulate hypoxia-induced factor-1 alpha (HIF-1α), which is critical to coordinate a local pro-inflammatory response (54). HIF-1α dimerizes with HIF-1β and translocates to the nucleus to modulate transcription of genes with promoters containing HIF response elements (HREs), with many of the induced gene products supporting the recruitment, retention, and function of pro-inflammatory macrophages. The expression of HIF-1α is essential to myeloid cell transition to glycolysis during pro-inflammatory immune responses. Early studies by Cramer et al. demonstrated that HIF-1α deletion using a lysozyme 2 (*Lys2*, or L*ysM*)-driven Cre recombinase resulted in monocytes and macrophages that were defective in glycolysis and, as a result, impaired their capacity for motility, invasiveness, and phagocytic ability (55).

The binding of TLR agonists by myeloid APC also shifts the cellular metabolism of myeloid cells towards glycolysis, which will supply ATP to support their inflammatory functions in oxygen sparse environments, but also generate nucleotides, lipids, and reactive oxygen species (ROS) used for anti-pathogen effector functions (56–59). Such metabolic changes originate from TLR ligation-mediated inhibition of mitochondrial oxidative phosphorylation (OXPHOS) and associated remodeling of the tricarboxylic acid (TCA) cycle. A pivotal determinant of myeloid cells’ metabolic reprogramming during the generation of a pro-inflammatory subset is the *de novo* expression of inducible nitric oxide synthase (iNOS). Expression of iNOS generates large quantities of nitric oxide (NO) that inhibits mitochondrial respiration through the stable nitrosation of Complex I of the electron transport chain (ETC), as well as reversible inhibition Complex IV and isocitrate dehydrogenase (60). Induced changes in the TCA cycle result in the generation of metabolic intermediates that are determinants of the macrophage inflammatory phenotype due to their enforced reliance on glycolytic metabolism and preventing macrophages’ repolarization away from a pro-inflammatory macrophage subset (61). The O’Neill group established that TLR4 ligation limits glutamine-dependent anaplerosis, or the replenishment of TCA cycle intermediates, to cause elevated levels of succinate to reach levels causing HIF-1α stabilization resulting in augment production of IL-1β (62). Interestingly, hypoxia alone was a weak inducer of *Nos2* mRNA in myeloid APC but synergized with TLR3, TLR4, and TLR9 agonists to prevent HIF-1α-dependent upregulation of *Nos2* mRNA and iNOS protein (63). The stimulation of TLRs by DAMPs like HMGB1, S100 proteins, mRNA, and mtDNA released during or after IRI in the hypoxic graft is a dominant driver of the pro-inflammatory responses that lead to early graft injury and failure, as well as stimulation of alloimmune responses that cause acute and chronic SOTx rejection.

To date, TLRs and their pathways have proven challenging to antagonize early post-SOTx to limit early inflammation, but HIF-1α would seem like an attractive downstream target that could suppress myeloid cell pro-inflammatory activity by limiting glycolytic metabolism. However, limited pre-clinical studies indicated that non-specific targeting of HIF-1α might be detrimental due to important graft tissue protections provided by HIF-1α in stromal and parenchymal tissues. In an orthotopic tracheal Tx model, adenovirus-mediated HIF-1α gene transfer to the graft promoted repair of mouse airway allograft microvasculature and attenuated CR (64). This effect was due to the HIF-1α-dependent recruitment of recipient pro-angiogenic cells that contributed to repairing damaged airway microvasculature. HIF-1α delivery before Tx increased graft perfusion to decreased fibrosis and improve graft survival (64). Other studies have demonstrated the importance of protective signaling of HIF-1α in the stroma and parenchyma after heart and kidney IRI before transplant (65, 66). The deletion of HIF-1α in macrophages causes the decreased production of vascular endothelial growth factor (VEGF), which is an important stimulus for initiating repair and vascularization of tissues damaged by IRI. While the early activity of the HIF-1α-VEGF is vital for the initiation of tissue repair, the sustained activation of the HIF-1α-VEGF pathway may later contribute to CR (67). HIF-1α is a critical factor that shapes the immune response after IRI and Tx; effectively targeting it will require both myeloid cell-specific delivery and understanding whether the graft’s current conditions will require a HIF-1α antagonist or agonist.

Recently, Ochando and colleagues completed exciting studies where they used myeloid cell-targeting nanoparticles to deliver an inhibitor of mammalian target of rapamycin (mTOR), which blocked HMGB1-induced glycolytic reprogramming (10). When mTOR-targeting nanoparticles were combined with antagonism of the CD40-TRAF6 axis, the result was long-term, fully MHC-mismatch cardiac allograft acceptance (10). The power of mTOR in stimulating macrophage pro-inflammatory functions was also demonstrated when the Medzhitov group established that IL-10 regulates macrophage pro-inflammatory responses by limiting glycolysis through the induction of a potent mTOR inhibitor, DDIT4 (68). IL-10 has long been understood to generate reparative and regulatory myeloid cells that support transplant tolerance, yet the mechanism(s) by which IL-10 mediates such potent impacts on myeloid cells has remained poorly understood. These studies established that myeloid cells stimulated with IL-10 resists the typical metabolic reprogramming induced by TLR4 ligation and, instead, maintained mitochondrial integrity and function to support OXPHOS. IL-10 also limits activation of the inflammasome by ATP in TLR4-stimulated macrophages. These recent investigations provide compelling evidence that myeloid cell-specific inhibition of mTOR and glycolysis will be an effective way to antagonist multiple DAMP pro-inflammatory pathways early after Tx.

ATP, while the energy currency of immunometabolism, has also emerged as a highly influential TLR-independent DAMP released by damaged, dying, and activated cells (6), particularly after Tx. Recent basic discovery and clinical studies have elucidated how immune cells release ATP as an inflammatory signal in response to allogeneic Tx and signal in a feedback mechanism *via* P2X7 to promote the release pro-inflammatory cytokines supporting rejection (17). The pro-inflammatory cytokine IL-1β plays a crucial role in early immune responses to tissue injury and pathogens, but even when induced by pro-inflammatory stimuli it is generated in an inactive pro-form that requires processing by caspase-1 to an active form. Activation of caspase-1 relies on the inflammasome, a multi-protein complex containing members of the nucleotide-binding domain- and leucine-rich repeat-containing receptor (NLR) family. The best-characterized inflammasome is NLRP3, whose activity is induced by a wide range of diverse stimuli, including PAMPs, numerous DAMPs, such as ATP and mtDNA, ROS, and even particulate matter (69). Activating the NLRP3 inflammasome and subsequent secretion of IL-1β requires two signals (69). First, a NFκβ activating signal, often a TLR-agonist, that induces and increases the expression of pro-IL-1β and NLRP3. A second signal, like the activation of the P2X7 receptor by extracellular ATP, or TLR detection of released mtROS or mtDNA, will cause inflammasome formation. The inflammasome activates associated caspase-1 that mediates the processing of pro-IL-1β or the closely related IL-1 family member, IL-18, into their mature and active cytokine form. Targeting P2X7 with an irreversible antagonist for 14 days after fully mismatched murine heart transplant promoted long-term cardiac transplant survival (18). Additional recent studies convincingly revealed that extracellular ATP is an early DAMP released by the transplant. ATP acts in a feed-forward loop to sustain high extracellular ATP levels in the graft by causing infiltrating recipient myeloid cells to release ATP locally. These high levels of ATP are crucial for augmented *Nlrp2, Casp1*, and *Il1b* expression in the graft, as well as, the secretion of IL-18 that contributes to type 1 alloimmune responses (17). While the survival benefits provided by inhibiting P2X7 in a rigorous skin transplant model was modest (17), the emerging importance of ATP and P2X7 after Tx suggest that targeting this pathway may be highly effective when combined with low doses of immunosuppression, or act synergistically with TLR antagonists to limit the activating signals feeding inflammasome/caspase-1 activation.

## Natural Pro-Inflammatory DAMP Regulators

As discussed above, the bulk of past Tx-related studies have sought to identify how to blunt DAMP pathways that initiate early inflammation after IRI responses with the prediction that this would lead to reduced alloimmune response or even aid tolerance induction. This approach has shown promise, particularly in experimental animal studies, where DAMP targeting with antibodies to DAMPs or their receptors reduces alloimmunity and limits AR. DAMP targeting also limited later development of CR-associated graft vasculopathy and fibrosis. Recently, another promising approach has been the identification of natural/endogenous pro-inflammatory DAMP regulators (**Table 2**) that the body utilizes to regulate pro-inflammatory DAMPs and harness them to limit alloimmunity or improve transplant outcomes. Past studies by Liu and colleagues demonstrated that the sialic-acid–binding immunoglobulin-like lectins (Siglecs)-CD24 signaling pathway suppresses inflammation triggered by DAMPs to protect against pathological inflammatory responses arising from cell death and necrosis (70). Importantly, they revealed that the Siglec-CD24 pathway only regulated DAMP signaling, while leaving the protective immune response to pathogen-derived PAMPs unabated (70). CD24 associates with DAMPs, particularly HMGB1, to negatively regulate their stimulatory activity by binding and presenting them to Siglecs that then downregulate immune responses *via* intracellular immunoreceptor tyrosine-based inhibitory motifs (ITIMs) domains (71). Active CD24-DAMPs-Siglec axis limits the inflammatory signaling in myeloid antigen, especially DC, to blunt their pro-inflammatory functions, particularly the secretion of TNF-α, IL-1β, and IL-6 (71). Harnessing this pathway has been particularly promising as a means to limit alloimmunity (72, 110) and assessment of the CD24-DAMPs-Siglec axis as a way to limit AR and CR after SOTx may also be worthy of further focused investigation.

**Table 2:** Regulatory or reparative DAMPs and related molecules in Tx.

Family	Molecule	Receptors	Role in Tx- related inflammation/immunity/outcomes	References
Regulatory or reparative DAMPs				
	CD24	Siglec	Associates with DAMPS to negatively regulate their stimulatory activity Protect against pathological inflammatory responses arising from cell death and necrosis Limits T cell alloimmunity	(70–73)
	IL-33	ST2	Promotes the systemic expansion of ST2^+^ Treg able to limit alloimmunity Promotes the secretion of Areg and other growth factors act on tissues and stem cells to support repair Induces TCR-independent Treg secretion of IL-13 and Areg that to control local inflammation and the generation of reparative type macrophages Directly promotes the generation of reparative macrophage phenotype through a metabolic reprogramming that augments OXHPOS and FA uptake	(74–82)
	Heat Shock Proteins (HSPs)	CD91, TLR2, TLR4, SREC1, and FEEL1	Supports debris clearance and wound repair Protect organs from IRI Extend graft survival Induce IL-10 secretion by T cells Support polarization of macrophages towards regulatory and reparative subsets	(24, 83–89)
	Hyaluronan	Lyve1	High weight forms support the survival and localization of macrophage subsets that productively remodel ECM to support vasculature function after injury Contribute to tissue integrity and functional immunological niches	(23, 90–94)
Specialized pro-resolving mediators (SPMs) and related molecules				
	Annexin A1	FPR2/ALX	Polarization of macrophage towards a pro-reparative subset Prolong allografts survival with sub-therapeutic immunosuppression Protect organs after IRI	(95–99)
	Maresins, Lipoxins, and Resolvins	GPR32 and ALX/FPR2 receptors	Limiting neutrophil infiltration and induction of neutrophil apoptosis Directly limiting adaptive immune responses Organ-protective and regenerative actions after IRI Stimulate macrophage transition toward reparative subsets Enhance Treg functions Prolongation of allograft survival	(100–109)

## Immune Cells Involved in a Regulated, Immune-Mediated Tissue Repair Process

### Tissue-Resident and Type 2 Cytokine-Activated Monocyte-Derived Macrophages

**Figure 1:**
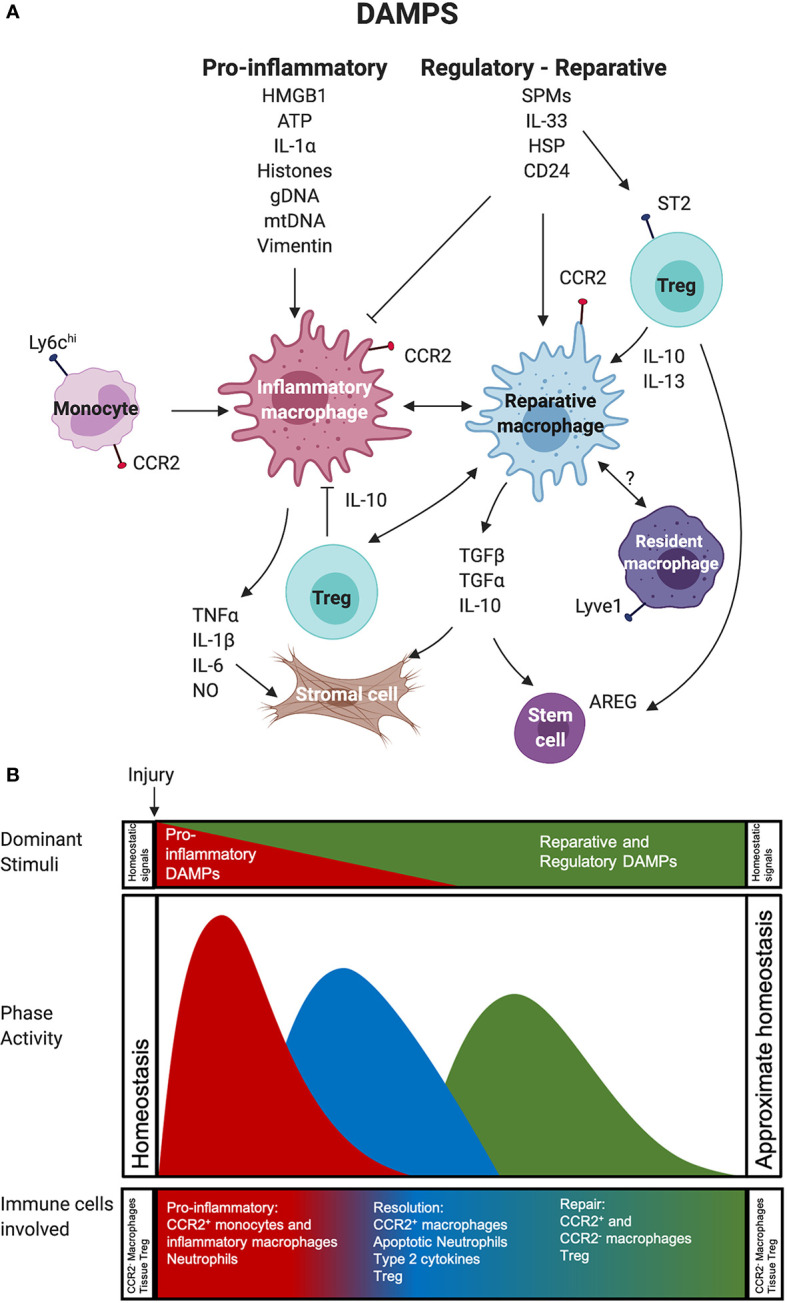
Impact of pro-inflammatory versus reparative and regulatory DAMPs on immune cells during inflammation, resolution, and repair phases after tissue injury. **(A)** Recruited CCR2^+^ monocytes and the macrophages derived from them participate in a highly regulated, immune-mediated tissue repair process shaped by *1.* Pro-inflammatory and *2.* Reparative and Regulatory DAMPs. These also act on Treg and potentially resident macrophages to support the survival and function of these immune cells. **(B)** This process can be divided into three overlapping phases, including a: *1.* pro-inflammatory phase (red), *2.* resolution phase (blue), and *3.* repair phase (green). In the first phase, pro-inflammatory DAMPs act on monocytes and macrophages to generate or support the function of highly phagocytic, inflammatory macrophages that use robust production of NO and pro-inflammatory cytokines to mediate the removal of any pathogens and damaged necrotic tissue. The transition to the resolution phase involves efferocytosis, or the phagocytosis of apoptotic cells, by macrophages receiving input from reparative and regulatory DAMPs and type 2 cytokines. These can both block the impact of pro-inflammatory stimuli on macrophages and contribute to the generation of Treg that support local immune suppression. The final phase involves little pro-inflammatory DAMP activity. It is dominated by reparative and regulatory DAMPs macrophage metabolism enabling the function of reparative and regulatory macrophages, such as secretion of cytokines, effector molecules, and growth factors that mediate responses in stromal, parenchymal cells, and stem cells to facilitate tissue repair. Regulatory and reparative DAMPs also act on Treg, which support the generation of reparative and regulatory macrophages and contribute growth factors to the repair environment. Reparative and Regulatory DAMP most likely also act on resident macrophages that are important for injury resolution and re-establishment and maintenance of tissue homeostasis. Abbreviations used: Areg, Amphiregulin; ATP, Adenosine Triphosphate; CCR2, CC Motif Receptor 2; DAMP, damage-associated molecular pattern; gDNA, Genomic DNA; HSP, Heat Shock Protein; HMGB1, High-mobility group box 1; IL, Interleukin; Ly6C, Lymphocyte antigen 6 complex, locus C1; mDNA, Mitochondrial DNA; NO, Nitric Oxide; SPM, Specialized pro-resolving mediators;TGF, Transforming growth factor; Treg, Regulatory T cell.

Tissues and organs of the body have resident populations of macrophages seeded during the embryonic or early postnatal period from early hematopoietic progenitors from the yolk sac and fetal liver, as well as a small subset originating later from circulating monocytes (111). In homeostatic mouse and human hearts, myeloid cells can be fractionated by their expression of the C-C chemokine receptor 2 (CCR2). At homeostasis, both rodent and human heart contain predominantly fetal-derived CCR2^-^ MHCII^lo^ macrophages, and small subsets of monocyte-derived CCR2^+^ MHCII^hi^ macrophages, as well as CCR2^+^ MHCII^lo^ monocytes (112). However, after the ischemic injury of organs and tissues, infiltrating monocytes and monocyte-derived CCR2^+^ macrophages rapidly dominate damaged tissues where they initiate the pro-inflammatory response discussed above. Studies of the CCR2^-^ population have demonstrated the importance of this subset to limit adverse remodeling, but the propensity to be lost at sites of IRI (90). Studies of IRI in commonly transplanted organs, such as the heart, lung, liver, and kidney, have provided evidence for the existence of certain DAMPs that not only initiate inflammation but also, or instead, initiate and sustain tissue injury resolution responses and repair by infiltrating immune cells. The study of mucosal injury and repair suggests a similar evolution where homeostatic resident myeloid cells are rapidly outnumbered by infiltrating monocytes and monocyte-derived macrophages (113). Through these models (114, 115), we now have a framework paradigm of an effective inflammation resolution and repair processes after tissue insult due to ischemic stimuli, and we are beginning to understand the DAMP-influenced processes and pathways directing immune cell-mediated response after injury (**Figure 1**). Findings in these injury models will not be confounded by the unique immunological situation found in SOTx where adaptive and innate immune cells will respond to non-self, allogeneic graft components. In injury models, a highly regulated, immune-mediated tissue repair process that is shaped by DAMPs after injury has emerged. This process consists of a pro-inflammatory phase, a resolution phase, and a repair phase (**Figure 1B**). How anti-AlloAg responses impact the typical signaling induced by DAMPs during the cellular responses leading to the early inflammatory phase and resolution and reparative phase after ischemic injury remains poorly understood. These questions are beginning to be addressed in recent rodent Tx studies described below, as well as speculated on in the later sections of our review.

As discussed above, myeloid cells are primary sensors of early damage. Yet, how infiltrating monocytes and tissue-resident macrophages respond to early hypoxia and DAMPs released due to ischemia is quite distinct. Infiltrating monocytes will differentiate into macrophages activated by pro-inflammatory cytokines (TNFα, IL-1β, IFNγ, IL-6) and DAMPs (HMGBI, ATP, Genomic DNA/Histones, IL-1α) into highly pro-inflammatory cells that, with neutrophils, dominate the pro-inflammatory phase after IRI. After ischemic injury due to myocardial infarction (MI), however, the resident CCR2^-^ macrophage subset, while able to proliferate in non-damaged tissues, is lost due to anoxia and nutrient depletion (90). Thus, the ischemic areas are rapidly dominated by responding neutrophils and infiltrating Ly6C^hi^ CCR2^+^ monocytes, and F4-80^+^ Ly6C^hi^ CCR2^+^ MHCII pro-inflammatory macrophages. The pro-inflammatory state of differentiation and functional activity of these macrophages will be enhanced by DAMPs like HMGB1 and Vimentin and local type-1 cytokines, particularly IL-1β, IFNγ, and TNFα. As described by Braza et al., HMGB1 and Vimentin promoted a pro-inflammatory training of cardiac graft-infiltrating macrophages that secreted increased TNFα and IL-6 (10). These type-1 cytokine-activated macrophages approximate the well-characterized “M1” macrophages generated *in vitro* by exposing macrophages to LPS and IFNγ. They use their high phagocytic capacity, robust production of NO, and pro-inflammatory cytokines to mediate the removal of any pathogens and any damaged necrotic tissue. These macrophages will also have high levels of HIF-1α, facilitating their glycolytic metabolism and the release of pro-inflammatory cytokines and chemokines that attract and activate additional infiltrating neutrophils and monocytes. The result is the collateral damage of healthy tissue from the induction of this response; thus, the benefit of limiting the pro-inflammatory response after Tx is apparent.

Nevertheless, this pro-inflammatory process involving macrophages is essential to address pathogens and dead cells and crucial to the initiation of the resolution phase and subsequent repair phase (**Figure 1B**) of the wound healing responses (57, 59, 116). Indeed, if macrophages are depleted early after IRI injury, the overall inflammatory response is greatly diminished, yet this results in ineffective clearing of necrotic cells from the damaged site and leads to inefficient repair and regeneration (117). Thus, one lesson from these studies for the transplant community is not to seek the total absence of an inflammatory macrophage response after Tx, but instead encourage a restrained early response that is brief and limited in scope to not cause overwhelming tissue damage that leads to early graft failure or persisting tissue injury. The success of reagents like co-stimulatory blockade in tolerance induction may be due, in part, from their ability to limit the antigen-presentation function of myeloid, but not ablate myeloid cell injury resolution functions. A vital transition in local macrophages next occurs in ischemic areas where they assume a phenotype associated with immune regulation and wound-healing. This transition is orchestrated by macrophage efferocytosis, or the phagocytosis of apoptotic cells, especially neutrophils, in the absence of pro-inflammatory stimuli and the presence of the type-2 cytokines, IL-4 or IL-13 (118).

A wealth of knowledge regarding type-2 cytokine activated macrophages has been generated through the *ex vivo* study of macrophages treatment with IL-4. IL-4 augments fatty acid (FA) uptake and oxidative phosphorylation (OXPHOS) to supports macrophage regulatory and reparative functions (57, 61). The importance of FA uptake and β-oxidation in regulatory and reparative macrophage polarization has been controversial (119). Yet, the disruption of FA uptake through inhibitors or loss of the FA translocase CD36 in mice and humans limits the generation and function of immunosuppressive and regulatory myeloid cells (120, 121). Inhibition of this pathway blocks the IL-4-induced expression of crucial genes, including CD206, CD301, and RELMα that are functional phenotypic markers of reparative and regulatory macrophage (61). Macrophages programmed towards repair through efferocytosis and IL-4 secrete the anti-inflammatory cytokine IL-10. IL-10 will act in the local environment to support macrophage OXPHOS and preserve their respiratory capacity by facilitating the removal of dysfunctional mitochondria *via* mitophagy (68). Upregulation of peroxisome proliferator activated receptor γ (PPARγ) and PPARγ coactivator 1B is important for FA oxidation and mitochondrial biogenesis in IL-4-exposed macrophages (122, 123). In addition to IL-10, type 2 activated macrophages secrete TGF-β and express programmed cell death ligands to suppress local immune responses (116). Arginase 1 (Arg1) is also induced to generate ornithine from L-arginine to support tissue repair (124), but also generates metabolites that dampen T cell responses, including those of alloreactive T cells (125, 126). IL-4-activated macrophages aid injury resolution through the production of several growth factors, including platelet-derived growth factor, transforming growth factorβ1 (TGF-β1), insulin-like growth factor 1 (IGF-1), and VEGFα to promote cellular proliferation, blood vessel development, and attract and differentiate tissue fibroblasts into myofibroblasts (116). The capacity of reparative macrophages to control myofibroblasts that modulate the local ECM to initiate wound contraction and closure and direct re-vascularization makes them critical to the restoration of injured tissues and organs as close to a homeostatic state as possible (121).

The ability of type 2-activated macrophages to both repair tissue and suppress local T cell responses has made them an attractive target population in SOTx to support tolerance induction, as well as limit or potentially even reverse CR (127). Further research is necessary to understand what endogenous local molecules initiate or support monocytes’ transition to reparative and regulatory macrophages at the end of the injury’s pro-inflammatory phase. The signals that direct their reparative response to resolve damage and restore homeostasis or functionality to damaged tissues and organs will be important targets to define for the generation of new biologics for use in SOTx. With a clear picture of the stimuli that control both the initiation, magnitude, and length of the inflammatory and repair and resolution phases after injury comes the capacity to control the process through regulated delivery of agents directing the appropriate pathways at the correct time. The elucidation of these macrophage-mediated pathways in innovative transplant models will be vital. These studies should also explain how the typical process and pathways leading to effective repair in tissues and organs are impeded, augmented, or dysregulated by a persistent local immune response to AlloAg.

### Lyve1^hi^ Macrophages

While infiltrating monocyte-derived macrophages are the prevailing effectors during the immune response to ischemic injury, other immune cells have been identified that also contribute significantly to effective healing and remodeling after tissue damage. A recent paper has described a Lyve1^hi^ MHCII^lo^ CX3CR1^+^ CCR2^-^ interstitial macrophage subset in the vasculature adventitia of the lung, heart, fat, and dermis (128). Depletion of this subset before bleomycin-induced lung injury or isoproterenol-induced cardiac hypertrophy augmented fibrotic disease in both models (128). Comparison of the arterial Lyve1^+^ vs. Lyve1^-^ macrophages revealed that the Lyve1^+^ subset was enriched for genes involved in homeostasis and ECM remodeling, and their deletion result in ECM abnormalities causing lost vascular wall integrity and impeded blood flow (91). Further mechanistic investigations revealed that Lyve1^+^ macrophages bind to smooth muscle cells (SMC) *via* ECM interactions and shape artery tone and function by regulating ECM collagen deposition (91). As mentioned briefly above, studies by Dick et al. have shown that a similar population of fetal-derived, TIMD4^+^ Lyve1^+^ MHC^lo^ CCR2^-^ cardiac resident cells are lost at sites of IRI. These cells are rapidly replaced by CCR2^+^ monocyte-derived cells, some of which can take on a CCR2^-^ resident phenotype, but lack expression of *Lyve1* or *Timd4* (90). The TIMD4^+^ Lyve1^+^ resident macrophage subset also repopulates after loss through proliferation in the peri-infarct area and their depletion post-MI resulted in poor cardiac function (90). Data generated using the depletion of resident CCR2^-^ macrophages before syngeneic cardiac Tx established that the therapeutic benefit they provide after IRI is due, in at least part, to their capacity to inhibit CCR2^+^ monocyte recruitment (92). It is not entirely clear if monocyte-derived Lyve1^+^ CCR2^-^ subsets are as effective as the fetal-derived subset they replace over time. There is, however, accumulating evidence that Lyve1^+^ CCR2^-^ macrophages are essential for the healing after cardiac IRI and contribute to local homeostasis by directing the infiltration of other immune cells through modulation of local ECM.

### Tregs in Tissue Repair

CD25^hi^ forkhead box P3 (Foxp3)^+^ regulatory T cells (Tregs) are an essential endogenous population of CD4^+^ T cells that act as potent immunosuppressive cells to control autoreactive immune responses and limit tumor immunity. Tregs use multiple mechanisms for their immunosuppressive functions that limit the size and quality of other T cell responses. These mechanisms include the production of anti-inflammatory cytokines, such as IL-10, IL-35, and TGFβ, that act directly on T cells to suppress their expansion and effector functions, as well as promote their exhaustion and deletion. Tregs ample expression of CD25 allows them to sequester IL-2 from immunological microenvironments. The importance of their suppressive capacity was first made evident in the study of mice and humans with Foxp3^+^ mutations that caused aggressive and lethal systemic autoimmunity (129, 130). Ongoing clinical trials are attempting to harness the potent immunosuppressive capacity of Tregs as cell therapy and reduce autoimmune pathology, or ideally, restore lost tolerance in patients with Crohn’s Disease, Type 1 diabetes, and lupus (131). Based on rodent pre-clinical Tx studies’ successes where administered polyclonal or AlloAg-specific Tregs support Tx tolerance induction, more than 15 clinical studies have been recently completed or underway in SOTx.

In addition to preventing tissue injury by limiting collateral damage mediated by an unrestrained immune response, Tregs also secrete factors that support the proliferation and survival of stem cells. Tregs secrete amphiregulin (Areg), a bi-functional growth factor that supports stem cell proliferation and differentiation through actions on the epidermal growth factor receptor (EGFR) (132). Related studies have identified Tregs secretion of keratinocyte growth factor as an import signal for alveolar epithelial proliferation and regenerative alveologenesis (133). In addition to the ability of Tregs to shape the function of monocytes and myeloid APC through secreted molecules like IL-10 and TGFβ, they also express indoleamine 2,3-dioxygenase that catalyzes the degradation of tryptophan to limit the function of CD8^+^ T effector cells (134). Tregs also have the capacity, at least *in vitro*, to direct the polarization of monocytes towards macrophage populations exhibiting features of those exhibiting reparative and regulatory functions *in vivo*. When both mouse or human monocytes are cultured with Treg, they upregulate their expression of CD206 and Arginase 1, both functional phenotypic markers of reparative and regulatory macrophages, due to Tregs secretion of IL-10 and IL-13 (135). Tregs are also essential to limit the damage and support function after ischemic injuries to the heart and brain (136, 137). Thus, in addition to their canonical role in suppressing detrimental immune responses and maintaining immune homeostasis, Tregs participate in the repair of tissue damage.

## Regulatory and Reparative DAMPs and Specialized Pro-Resolving Mediators (SPMs) in Tissue Injury Resolution

In the above sections, we outline the importance of several macrophage subsets, with input from Treg, needed to complete a highly regulated resolution and repair process that is relatively universal across organs and tissues. While not the focus of this review, it should be mentioned that other immune cells, particularly dendritic cells, various T helper subsets, and innate lymphocytes, also play essential roles in the repair process initiated by DAMPs (3, 138). It is also hopefully more clear how pro-inflammatory DAMPs, like HMGB1, ATP, and mDNA, are important initiators of, or at a minimum - crucial contributor to the early pro-inflammatory phase of tissue injury. Nevertheless, other endogenous signals, like CD24, that quell local inflammation or initiate the resolution and repair phases after IRI or other Tx-relevant injuries remain poorly understood. Previously suggested pro-inflammatory DAMPs, particularly IL-33, HSPs, and HA, however, support the expansion of reparative cells. These DAMPs also drive the function of immune cells during the resolution and reparative programs induced in various injury models (**Table 2**). Other biomolecules, such as Annexin A1 (AnxA1) and specialized pro-resolving mediators (SPMs), including resolvins and maresins, also act as powerful endogenous signals that support immune-mediated inflammation resolution and the return to local homeostasis (**Table 2**).

When considered from a more general perspective, there are several common characteristics of regulatory DAMPs and SPMs that standout. First, these molecules are typically sequestered or shielded from recognition by the immune system until they are released after injury. Second, both groups limit local infiltration by inflammatory leukocytes and instead orchestrate the differentiation and function of immune cells that restore local homeostasis through inflammation resolution and repair. Third, most contribute to the differentiation of reparative- or regulatory-type macrophages *via* induced signaling and metabolic programming towards OXPHOS and FA uptake. Fourth, the capacity of regulatory DAMPs and SPMs to directly stimulate Treg expansion and function, or support Treg expansion indirectly through actions on myeloid APC and macrophages is common. Finally, many of these molecules may be released at very low concentrations during normal cell turnover to sustain the immune cells maintaining homeostasis. The molecules and immune cells, like Treg and macrophages, restoring local and systemic homeostasis, may overlap considerably with cells and systems that typically maintain it. We discuss below the limited, but growing, literature describing a potential role for regulatory DAMPs and SPMs and their target cells in influencing alloimmunity and Tx outcomes.

### Specialized Pro-Resolving Mediators (SPMs)

SPMs are a superfamily of lipid molecules that are generated locally after injury and target G coupled receptors (GPRs) in order to stop excessive neutrophil infiltration, counter pro-inflammatory signals, enhance efferocytosis, and the clearance of dead cells by macrophages (139, 140). SPMs are generated from essential polyunsaturated FA in enzymatic reactions completed by both leukocytes, platelets, and parenchymal and stromal cells into several related groups of immunoresolvins, including lipoxins, E, and D series resolvins, protectins, and maresins (100, 139, 140). Lipoxin is secreted by neutrophils and macrophages after being synthesized from arachidonic acid. It acts on cells expressing the G protein-coupled lipoxin A4 (ALX)/formyl peptide receptor (FPR2) or GPR 32, the aryl hydrocarbon receptor, estrogen receptor, as well as the cysteinyl leukotriene receptor (141). Important actions of lipoxin on innate immune cells after injury include limiting neutrophil infiltration and the induction of neutrophil apoptosis. Lipoxin also supports injury and inflammation resolution by delaying the apoptosis of macrophages completing efferocytosis and local debridement (142). Limited studies suggest that lipoxin may have the ability to directly regulate B-cell antibody production and proliferation, as well as limit T cell effector functions (101, 102). While lipoxin impacts on innate and adaptive immune cells would be expected to improve Tx outcomes, to date, however, the influence of lipoxin on alloimmunity and Tx outcomes has been poorly explored. A limited assessment in clinical lung Tx samples revealed the presence of lipoxin in these samples, and the delivery of a stable lipoxin analog provided subtle improvements in mouse heart and kidney Tx models (103). This initial testing was completed in MHC-fully mismatched models; thus, experimentation in less aggressive combinations is warranted to understand better if lipoxin can improve CR by limiting IRI, reducing alloimmunity, or initiating repair responses.

E-series resolvins are generated primarily by neutrophils from the exudate omega-3 FA eicosapentaenoic acid (EPA), where D-series resolvins are made by neutrophils from docosahexaenoic acid (DHA), which also serves for the starting blocks of maresins, which is synthesized from DHA by macrophages (100). Like lipoxin, both resolvins limit neutrophil infiltration and promote their apoptosis. Some resolvins have potent organ-protective and regenerative actions that would be highly relevant in surgery-induced IRI. SPM-stimulated macrophage transition toward those reflective of IL-4-activated macrophages, which, as discussed, are characterized by high levels of FA uptake and are the primary source of maresins (104). These lipid mediators have also been shown to induce macrophage production of IL-10 while reducing dendritic cell production of IL-12 (100, 105). SPMs also have potent anti-IRI activities demonstrated for kidney, liver, and lung mediated by limiting TLR4/MAPK/NF-κB pathway activity and activating the Nrf2 pathway to limit oxidative stress (106–108). Other intriguing studies have suggested that D-series resolvins and maresin can act on T cell GPR32 and ALX/FPR2 receptors to limit human and mouse pro-inflammatory cytokine production, while simultaneously enhancing Treg function. It has also been described how a decrease in resolvins and maresins are observed in obese subjects or individuals suffering from autoimmunity or systemic inflammatory diseases. These findings suggest the importance of these molecules in systemic homeostasis (143). Based on these effects, the role of SPMs in the early and late immunobiology of Tx deserves investigation.

### Annexin A1 (AnxA1)

AnxA1 is a phospholipid-binding protein sequestered in the cytoplasm of neutrophils, monocytes, and macrophages and released upon their activation (144). The production of AnxA1 is highly responsive to glucocorticoids, with endogenous and delivered glucocorticoids increasing both AnxA1 expression and secretion (144). The anti-inflammatory and pro-resolving effects of AnxA1 are mediated through binding to FPR2/ALX, which limits neutrophil transmigration tissue infiltration and induces neutrophil apoptosis. AnxA1 acts on macrophage FPR2/ALX receptors to activate AMPK, which is a potent regulator of mTOR (95). This results in the polarization of macrophage towards a pro-reparative subset. These data indicate that AnxA1 acts as a natural factor that can regulate the pro-inflammatory DAMP metabolic reprogramming described by Braza et al. Thus, AnxA1 may act like the pro-tolerogenic signals generated when nanoparticles containing the mTOR inhibitor rapamycin were used targeted to graft macrophages after heart transplant (10). Delivery of an AnxA1 mimetic could prolong BALB/c skin grafts on B6 recipients, but only when given with sub-therapeutic cyclosporine A (96). Targeting FPR2/ALX with AnxA1, like lipoxin above, provides a protective, but not robustly immunosuppressant or protective effect after Tx. Given the importance of limiting early graft injury and rapidly transitioning from a local pro-inflammatory state to one of injury resolution and tissue repair, it is easy to envision how reagents targeting this pathway could be combined into immunosuppressive protocols to improve outcomes by limiting the pro-inflammatory phase and accelerating pro-inflammation resolution after IRI. Tx researchers have spent most of our energy looking for reagents that are potent immunosuppressants or able to induce tolerance. AnxA1 may be able to contribute here through actions on Dectin-1 (145). However, it is advisable that we also harness the wealth of past evidence that AnxA1 or its derivatives are useful when used to target FPR2/ALX to limit MI-mediated pathology and acute kidney injury (97–99). These data would support an investigation into using these reagents to limit early graft failure or IRI under cover of immunosuppression.

### Heat Shock Proteins (HSPs)

Heat shock proteins (HSPs) are highly conserved proteins grouped according to their molecular weights (e.g., Hsp 27, Hsp40). They are upregulated in response to stress conditions that result in damaged proteins, such as extreme heat, hypoxia, oxidative stress, inflammation, injury, or infections. They are essential for intracellular functions involving the initiation of protein folding, repair, refolding of misfolded peptides, and aiding in the degradation of irreparable proteins. However, upon necrotic cell death or cellular stress, HSPs are released and were initially characterized as pro-inflammatory DAMPs that acted on TLR4. However, several pre-clinical studies have demonstrated various extracellular HSPs that, when overexpressed or delivered, can protect organs from IRI (83, 84) and extend graft survival (85, 86). When delivered, various HSPs are potent inducers of T cell IL-10 production and support the polarization of macrophages towards regulatory and reparative subsets (87, 88). These findings and their implications in Tx have been recently expertly-reviewed (89) and thoroughly describes the literature supporting consideration of HSPs as a regulatory DAMP in Tx.

### Hyaluronan (HA)

HA, also known as hyaluronic acid, is an important ECM component synthesized by HA synthase at the plasma membrane of predominantly mesenchymal cells. Here it associates with different HA-binding proteins to form pericellular and extracellular matrices that are important for creating space and a matrix allowing cellular migration and localization. HA is also generated as part of the tissue injury and repair response, where it directs and regulates the infiltration and function of fibroblasts, blood vessels, and immune cells (93). HA is connected to the early inflammatory responses after injury, as degraded HA induces signaling *via* TLR4 and TLR2 on macrophages to drive pro-inflammatory cytokine production (21). HA has a long history in Tx, as Goldstein and colleagues showed convincingly that HA fragments could induce DC maturation and initiate alloimmunity (22). That HA can induce alloimmunity is of clinical relevance as the bronchial lavage fluid of lung transplant patients undergoing AR displays significantly higher HA levels than those with no rejection (146). Additional studies established that HA was prominent in areas of intraluminal small airway fibrosis in lung transplants bronchiolitis obliterans, as was the message for HA synthase (147). Increased local and circulating levels of HA have also been noted in rodent skin and cardiac transplant models (22, 148). Yet, the impact of HA on alloimmunity is not clear and potentially double-edged, as different molecular weight HA products seem to produce pro-inflammatory or regulatory impacts depending on the transplanted organ. The accumulation of lower molecular weight HA stimulated lung inflammation after lung injury and was shown to contribute to lung transplant rejection, while high-molecular-weight HA attenuated allograft inflammation and contributed to lung epithelium integrity (21–23, 147). In contrast, low molecular weight HA delivery prolonged renal and cardiac allograft survival (149, 150). These contrasting findings may reflect different biological functions of HA between different organs and or distinct roles of HA in the different physiological processes happening, i.e., AR, CR, or tissue repair.

One aspect that should be discussed further that may account for these varied responses in transplanted organs is the emerging importance of intact, high molecular weight HA to the generation of functional immunological and repair niches. HA interactions are critical to hematopoietic and tissue-forming stem cell migration, function, and survival (93). HA stem-supporting characteristics also aid the function and survival of cancer cells, and HA in the ECM of the tumor environment supports tumor-associated macrophages’ polarization and survival (151, 152). Thus, while the DAMP activity of HA is an important consideration, an equally important function of HA in the transplant microenvironment may be its role in providing the localizing, supporting structure to the hematopoietic and structural cells that are being tuned by DAMPs and other signals in the environment to shape any alloimmune response or resolve a local injury. HA contributions to “rejection” niches are implied by the observation that delivery of low molecular weight fragments antagonize cardiac graft infiltration by effector cells using the HA binding receptor CD44. This interaction can also be targeted effectively with anti-CD44 antibodies (153).

Nevertheless, several recent studies have revealed the importance of HA niches and protective HA-binding myeloid cells in them after IRI. As introduced above, Dick et al. described the importance of fetal-derived, self-renewing Lyve1^+^ MHCII^lo^ CCR2^-^ macrophage subset in productive repair after myocardial infarction (MI) (90). Lyve1 is the receptor for HA, and the expression of this receptor by CCR2^-^ macrophages appears to target them to HA dense areas, particularly the adventitial layer of arteries. Deletion of these macrophages post-IRI resulted in the dysregulated repair after myocardial infarction (90). Related studies also used a different system to completed targeted deletion of Lyve1^+^ macrophages and established that an important function this subset was to modulate the ECM in these arterial niches and prevent arterial stiffness at homeostasis (91). This function required Lyve1-HA-interaction-induced production of matrix metalloproteinase 9 (91). Further mechanistic investigations revealed that Lyve1^+^ macrophages bind to smooth muscle cells (SMC) *via* interactions with HA and shape artery tone and function by regulating ECM collagen deposition (91). HA’s importance for healing after IRI was also demonstrated in mice with inducible deletion of HA synthase 2 (HAS2). HAS2 deletion before IRI resulted in a severely impaired hemodynamic function associated with a loss of cardiac macrophages, but not monocytes (94). The authors accounted poor function to increased apoptosis of macrophages in the absence of HA stimulation (94). The loss of HA also resulted in decreased myofibroblast in the infarct site, and *in vitro* studies outlined an intricate network were HA-positive fibroblasts and Lyve1^+^ macrophages communicate to generate functional ECM after IR. These observations mesh with syngeneic cardiac Tx studies completed by Kreisel and Lavine, where they demonstrated that CCR2^-^ macrophages inhibit monocyte recruitment, where the CCR2^+^ macrophage subset promoted monocyte recruitment *via* MyD88-dependent mechanism and the release of monocyte chemoattractant proteins (92). These recent studies shed light on how critical local niches shaped by ECM components, including HA, will be important to outcomes after Tx.

### Interleukin-33 (IL-33)

IL-33 is a member of the IL-1 superfamily sequestered in the nucleus due to a nuclear localization domain and chromatin binding motif (154, 155). IL-33 released during necrotic cell death and cellular stress is functional, but its activity is negatively regulated by caspases, oxidation, and chromatin occupancy. Several proteases can increase the activity of full-length IL-33 by cleaving off the nuclear localization domain and chromatin binding motif (154, 156). We have also recently demonstrated that bio-active IL-33 is present in vesicles bound to the ECM of stromal cells where it is protected from proteolytic modification (157).

IL-33 was originally identified and described as inflammatory DAMP that drives type 2-cytokine-mediated inflammation when it is released after tissue damage and stimulates immune cells *via* the IL-33 receptor IL-1R-like-1(IL1RL1), more commonly referred to as Stimulation-2 (ST2) (158). Numerous immune cells express varying levels of ST2. These include basophils, mast cells, eosinophils, group 2 innate lymphoid cells (ILC2s) (154), CD8^+^ (159, 160), and CD4^+^ T cells (161), particularly Th2 cells and Treg (74–77, 158, 162), B cells (163), macrophages (78, 157, 163), and DC subsets (162, 164, 165). IL-33 acts on these cells to support type 2 responses dominated by the cytokines IL-5 and IL-13. IL-33 induction of type 2 cytokines aides parasite clearance and drives allergic responses, lung inflammation, and fibrotic skin diseases. There is a close link between type 2 cytokines and tissue repair, and IL-33 has emerged as a crucial mediator of the repair process. Much of the known repair activity of IL-33 involves its capacity to target ST2^+^ Tregs, a predominantly peripheral tissue-resident subset, and induce their expansion and production of IL-10, IL-13, and Areg (166). Seminal studies by the Rudensky group established an essential role for Tregs in the resolution of epithelial injury after virally-induced lung injury due to their secretion of Areg. Interestingly, it was Tregs recognition of IL-18 or IL-33, not TCR signaling, that led to this reparative action (74). IL-33 also induces TCR-independent Treg secretion of IL-13 that is critical to control local inflammation and after chemical or viral lung injury (77). Treg secreted IL-13 generates Arginase 1^+^ macrophages implicated in tissue repair and homeostasis (77). ILC2 secrete IL-13 in response to IL-33 to promote lung regeneration by stimulating macrophage support of type 2 alveolar epithelial stem cell proliferation (167). There is a prominent role for IL-33 in regulating metabolic homeostasis, and disruption of the Treg-ILC2-Macrophage axis contributes to increased inflammation and obesity (168–170). Fibro/adipogenic progenitor cells in the skeletal muscle express IL-33 and sustains skeletal muscle Tregs that are important for muscle regeneration after injury through secretion of Areg that supports muscle satellite cells (75, 76) and potentially limits the local generation of inflammatory Ly6C^hi^ macrophages (75).

Numerous studies have suggested the potential to harness the emerging regulatory and reparative properties of IL-33 in Tx. Administration of IL‐33 post-heart Tx expands ST2^+^ Treg to prolong allograft survival across MHC barriers in rodent heart transplant models (79, 158). Skin graft acceptance could also be aided through IL-33-induced expansion of regulatory myeloid cells and Treg (80, 81). It was not until recently that we also revealed an essential regulatory function for graft-derived IL-33 that involved the direct targeting of infiltrating recipient monocytes and macrophages (78). We used heart transplants lacking IL-33 or recipients with ST2-deficient macrophages to clarify that a critical function of endogenous IL-33 was to promote the generation of reparative macrophage phenotype through a metabolic reprogramming augmenting OXHPOS and FA uptake. Thus, IL-33 is unique relative to DAMPs like HMGB1 that drives glycolysis and epigenetic modifications enabling inflammatory cytokine production (10). IL-33 instead blocks iNOS expression and, like IL-4, IL-10, and IL-13, increases mitochondrial function and FA uptake (61, 78). In total, it is safe to describe IL-33 as a regulatory DAMP in Tx, and it will be necessary to use tissue-specific disruption of IL-33 and immune cell-specific deletion of ST2 to help us further understand how IL-33 coordinates responses to IRI and alloinjury after SOTx.

## Understanding Where Pro-Inflammatory and Regulatory and Reparative Damp Signals Get Tangled and Lead to Poor Outcomes After Tx

As outline above, the process of injury recognition, inflammation initiation and resolution, and then tissue repair after IRI is complex in both signals and cells involved. It is also subject to pathology when not perfectly orchestrated, or a phase in the process is amplified or incomplete. It is easy to appreciate how an augmented inflammatory response due to an extended ischemia period releasing prodigious amounts of pro-inflammatory DAMPs across an entire organ can lead to early graft dysfunction and failure. It is clear how this would also lead to AR due to widespread activation of resident DC and other APC presenting AlloAg, which then travel to the secondary lymphoid organs to stimulate an alloimmune response. The inflamed tissues would also be an ideal environment for the generation of inflammatory APC as infiltrating recipient monocyte differentiate into pro-inflammatory macrophages and DC that support local alloresponses that drive rejection (171). The ongoing efforts discussed above to block the early inflammation mediated by pro-inflammatory DAMPs, if found therapeutic, should have an impact here. However, despite the availability of potent immunosuppressants available to target adaptive immune responses and the shortening ischemia times common in current clinical transplant medicine, CR remains a persistent problem. The development of CR in immunosuppressed individuals suggests that other factors beyond pro-inflammatory DAMPs may need to be considered. In this remaining section, we briefly postulate how unique aspects of SOTx may interfere with appropriate resolution or re-establish tissue homeostasis after Tx to lead to CR.

### Alloimmunity Prevents Effective Resolution and Repair

**Figure 2:**
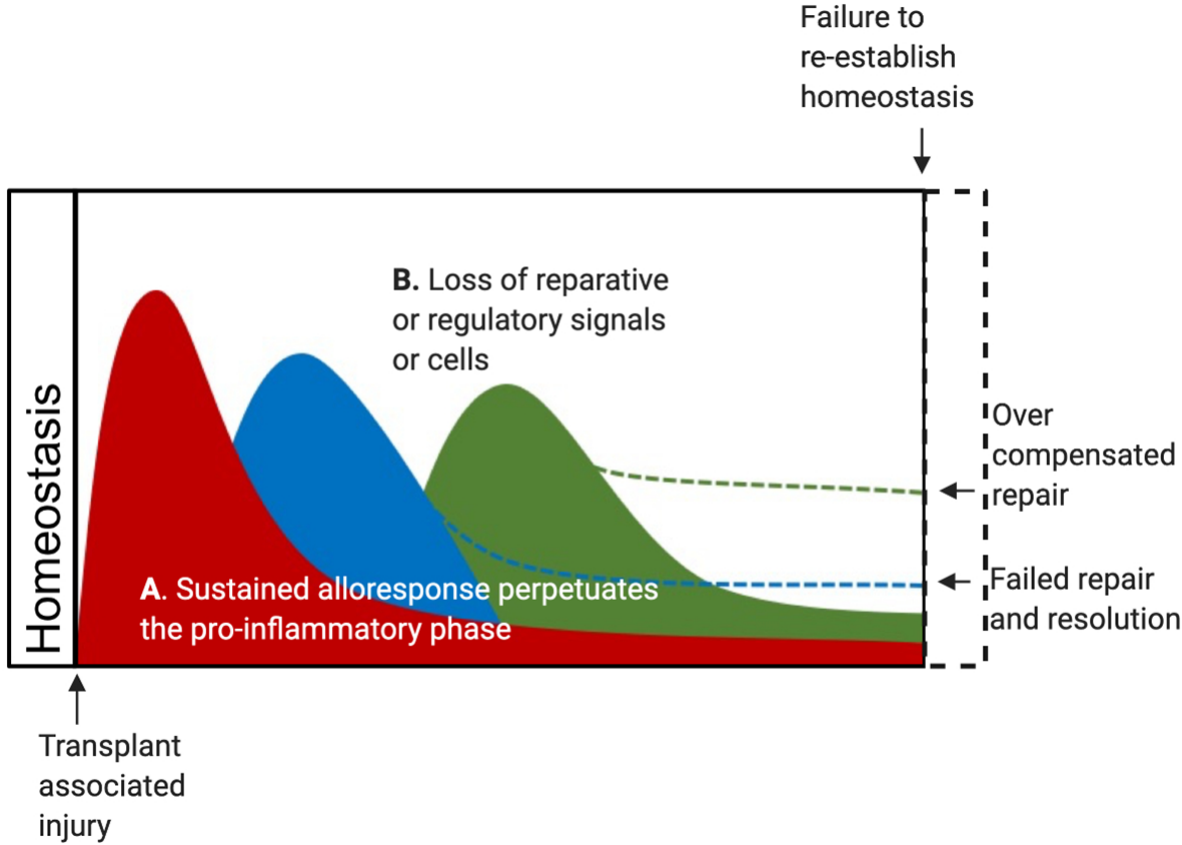
DAMP may contribute to CR after Tx in several scenarios. **(A)** The alloreactive response of innate and adaptive cells may sustain the pro-inflammatory phase and lead to a failure to fully transition through the resolution phase and complete the repair phase. In this scenario, CR represents a failure to resolve and repair, leading to residual graft damage and failure to restore tissue homeostasis. Alternatively, these changes may cause an overzealous counter-responses initiated by reparative Treg and reparative macrophages in responses to regulatory and reparative DAMPs released by an unresolved allogeneic wound. **(B)** Alternatively, lost or depleted reparative and regulatory cells due to ischemia or alloimmune responses may lead to a failed resolution phase leading to residual graft damage. This scenario may also arise from a depletion of local reparative and regulatory DAMPs over time. In this case, CR would represent a failure to restore tissue homeostasis due to persisting graft damage.

Transplanted organs represent a unique immunological situation where non-self, allogeneic signals will impact the typical immune responses working toward resolving early ischemic injury and any damage caused by allorecognition. The reaction to AlloAg by the adaptive immune systems, as well as NK cells, has been long recognized, yet how innate alloimmune responses influence acute and chronic tissue injury resolution and repair responses remains unclear. Precise mouse studies have now established that graft infiltrating monocytes, in addition to detecting DAMPs, will recognize allogenic molecules, such as the polymorphic signal regulatory protein α (SIRPα). The binding of allogeneic SIRPα to the nonpolymorphic CD47 causes monocytes to mature into monoDCs expressing IL-12 and stimulating T cell proliferation and IFNγ production in the graft (172, 173). Murine monocytes and macrophages can also recognize and acquire memory specific to MHC-I antigens *via* paired immunoglobulin-like receptors-A (PIR-A) (174). As outlined in **Figure 1**, these infiltrating monocytes are the main coordinators of local DAMP and cytokine signals needed to initiate and then resolve tissue injury. It is easy to speculate how alloreactive macrophage will increase local IL-12 and IFNγ to prolong the pro-inflammatory phase or prevent transition to resolution and repair (**Figure 2A**). Nevertheless, these changes may augment counter-responses to increase damage and regulatory and reparative DAMPs (**Figure 2A**). Thus, CR may instead result from an overzealous or persistent resolution response mediated by reparative Treg and macrophages that sustain a response to an unresolved allogeneic injury.

### Replacement of Donor CCR2^-^ Macrophages by a Recipient CCR2^+^ Pro-Inflammatory Subsets

The importance of fetal-derived, Lyve1^+^ CCR2^-^ resident macrophages cells to control pro-inflammatory CCR2^+^ monocytes’ infiltration and mediate ECM remodeling to allow inflammation resolution and productive tissue repair after cardiac IRI was laid out above. Comparable populations of self-replicating, fetal-derived macrophages are noted around the vasculature of the lung, fat, dermis (128), as well as in the kidney (175). The CCR2^-^ macrophage subset appears critical for local control of inflammation and remodeling after injury, but susceptible to loss due to conditions typical of IRI and CR, including hypoxia and the loss of an HA-rich ECM. Once lost, this subset is rapidly replaced by a circulating CCR2^+^ monocyte-derived subset, which lacks the capacity of the CCR2^-^ subset for limiting fibrosis (90–92). Similar findings in human transplant samples were observed when endomyocardial biopsies from sex-mismatched heart transplant recipients were assessed for the presence of donor tissue-resident CCR2^-^ macrophages (112). These studies confirmed that the CCR2^-^ subset was almost exclusively donor-derived. Parallel transcriptomic profiling of CCR2^-^ and CCR2^+^ macrophages isolated from failing human hearts were consistent with the conclusion that these two populations were distinct cell types. The CCR2^+^ subset appeared monocyte-derived and expressed inflammatory mediators, including IL-1β, components of the inflammasome, and genes involved in adverse cardiac remodeling. The CCR2^-^ subset instead expressed increased Lyve1, growth factors, and ECM genes implicated in tissue remodeling. The CCR2^+^ macrophage subset was more abundant than the CCR2^-^ subset in heart failure samples from those with worse left ventricular systolic dysfunction and adverse remodeling (112). This seminal study provides the initial confirmation of a potentially beneficial donor CCR2^-^ macrophage population in transplanted organs.

Further studies can build on this work to define if shorter ischemia times, *ex vivo* normothermic perfusion, or specific immunosuppression protocols can prevent or slow the loss of donor CCR2^-^ macrophages and the subsequent replacement by CCR2^+^ recipient macrophages after SOTx. These examinations or related pre-clinical studies will provide an understanding if AR, CR, IRI, or recipient alloimmunity causes the CCR2^-^ subset to drop below a significant reparative threshold after Tx. It would be expected that this would lead to increased inflammation and a sustained loss of homeostasis (**Figure 2B**). Such a scenario would account for increased alloimmunity and fibrosis, that culminates CR pathology after SOTx (**Figure 2B**). An important additional question to answer if local reparative DAMPs support the survival or local proliferation of the CCR2^-^ subset during homeostasis or after IRI.

### Dysregulated Local Niches in the Tx Microenvironment

Immunological niches typically provide a hospitable place that concentrates the signals needed to nurture the immune and stromal cells need to maintain an effective local immune response or local homeostasis. An organized immunological microenvironment, or niche, controls local immune responses during tumor development, is necessary for regulating immune cell functions in the secondary lymphoid organs, and fundamental to the production of blood cells in the bone marrow (176–178). We briefly discussed how HA-rich niches in the adventitia are critical for the homeostatic maintenance of vessel function and how these can be disrupted through the loss of specific cells like CCR2^-^ macrophages or local environmental signals like HA. The role of immunological niches in transplant outcomes is currently entirely speculative but is an exciting concept. However, based on what is known about both IRI and the alloimmune response after Tx, we would expect that “homeostatic,” “acute rejecting,” and “injured/reparative,” “dysregulated/fibrotic/CR” areas all would be observed, and often co-exist, throughout the lifespan of a transplant. Work in this space by the Halloran group revealed using unsupervised principal component analysis and archetypal analysis on microarray assessment of HTx endomyocardial biopsies (EMB) identified that samples that abnormal EMB did not associate cleanly with rejection and instead expressed transcripts indicating a tissue injury response (179, 180). These samples were enriched for transcripts for DAMPs, as well as macrophages. The injury-related scores were also high at early times post-transplant and routinely diminished over time. The decreasing rejection scores suggest that repair and resolutions of global IRI injury to the graft is indeed typical unless interrupted by a local alloimmune response that is not effectively inhibited by immunosuppression.

IL-33 has been implicated in adventitial vascular niches, where the IL-33 deletion causes an inadequate local immune response to pathogens (164). Ablation of IL-33 from white adipose niche caused immune dysregulation in these niches resulting in immune dysfunction and obesity associated with increased pro-inflammatory myeloid cells (181). A high-fat diet also reduces IL-33 expression in the white adipose niches to produce similar outcomes. As discussed above, we found that the upregulation of IL-33 during clinical and experimental HTx rejection decreased CR due to this regulatory DAMP’s potent capacity to limit the generation of pro-inflammatory macrophages from monocytes infiltrating the grafts (78). Nevertheless, it is yet to be determined how IL-33 or other regulatory DAMPs are maintained in SOTx regions of the graft with acute or sustained alloimmunity. Limited evidence from EMB suggests that grafts maintaining IL-33 display less CR (78). It is known that IL-33 decreases with age in the muscle leading to inadequate regenerative responses associated with decreased Treg and increased inflammatory macrophages. How the expression of these DAMPs are modulated in fibrotic areas to instruct local CCR2^-^ and CCR2^+^ macrophages will be an essential question to answer. A lost local repair response may become further augmented when niches become depleted of reparative, or regulatory DAMPs or the niche ECM becomes unsupportive of cells needed for repair and instead overtook by alloreactive T cells that are stimulated by pro-inflammatorymacrophages. As vessels become occluded due to damage or CR, the niche will become hypoxic and may further drive macrophages towards pro-inflammatory subsets supporting rejection. Conversely, sustained hypoxic environments in areas of the graft could instead favor the generation of regulatory macrophages due to the induction and modulation of local DAMPs. Both HMGB1 and IL-33 functionality is impacted by their redox state. While oxidation of IL-33 into a disulfide-bonded form negatively regulates its function (182), oxidized HMGB1 induces the expression of proinflammatory cytokines and chemokines by macrophages through its binding to MD-2 and TLR4 (183). Conversely, reduced HMGB1 associates with the chemokine CXCL12 and binds the CXCR4 receptor to recruit circulating leukocytes and stem cells to the site of damage and promote tissue regeneration (184, 185). Reduced HMGB1 in hypoxic tumor sites is suggested to generate regulatory and reparative macrophages that shape an immunosuppressive tumor microenvironment (186). HSPs would also be induced in hypoxic areas or by cell stress associated with ischemia, rejection, and fibrosis. Important future studies will be needed to establish how reduced, oxidized, or induced DAMPs function in hypoxic versus normoxic regions of solid organ transplants to dictate short and long-term outcomes.

## Conclusions

The original concept that DAMPs function after Tx as endogenous PAMPs in one-way paths that can be blocked to prevent early inflammation while regulatory and repair signals proceed unabated is dated. Our current understanding, generated from limited Tx data and studies of organ IRI models, is that the transcription factors and metabolic processes activated by pro-inflammatory DAMPs triggering inflammation after IRI and tissue injury are an essential part of a dynamic process that needs to function to trigger resolution and allow damaged tissues to return to homeostasis. It is also clear that regulatory and reparative DAMPs, closely related SPMs, are also significant players in shaping the ideal size and duration of the inflammatory phase after tissue injury. Regulatory and reparative DAMPs are also active mediators of subsequent resolution and repair phases.

As tools such as scRNAseq and spatial transcriptomics become more widely applied in Tx, it will become more apparent how these different subsets of DAMPs contribute to immune cell networks during effective responses to IRI and how these are altered by local alloimmune responses by innate and adaptive cells. Value-added histology approaches utilizing mapped total RNA analysis (i.e., 10x Genomics Visium technology) or multiplexing immunofluorescent tags detecting RNA messages or specific proteins (i.e., Nanostring GeoMx technology) will be incredibly helpful to add to our understanding of the DAMP-driven immunology and physiology existing throughout the graft. Chronological graft assessment will define how sustained generation or depletion of regulatory or reparative signals triggering inflammation, resolution, and repair are modulated in graft AR and CR in crucial spaces, such as the vasculature. Applying advanced bioinformatics techniques such as artificial intelligence and machine learning can be used to investigate immune cell/DAMP interactions to help establish how these interactions shape the active signaling networks at each step after injury, inflammation, resolution, and repair of the graft (187). Using these types of analyses with precise mouse models allows for temporal control of the local DAMPs or AlloAg, which will allow us to untangle AlloAg input into pro-inflammatory, resolution, and repair pathways after SOTx.

This knowledge will provide the transplant community with a framework for developing precision-medicine approaches where biologicals direct immune processes in the graft effectively. This family of drugs will be delivered to modulate dominant networks active in the graft instead of typical efforts to target individual immune populations or cytokines. Given the emerging evidence that DAMPs are important mediators of both early inflammation, injury resolution and repair there is significant therapeutic potential in manipulating the expression or delivery of DAMPs during the course SOTx. We have shown that the delivery of regulatory biomolecules, such as IL-33, using a hydrogel immediately post transplantation could improve outcomes by reducing the generation of inflammatory macrophages in HTx early after transplantation (78). Exploration into the *ex vivo* manipulation of organs prior to transplantation as a means to minimize inflammation and induce the expression of regulatory DAMPs is warranted. With the more recent development of normothermic *ex vivo* organ perfusion storage to mitigate IRI, there is a window of opportunity to biologically modify the donor organ either through the delivery of biomolecules, including regulatory DAMPs, encased in biovesicles or synthetic nanoparticles or potentially through gene therapy (188, 189). Another potential therapy to investigate is hypoxic pre-conditioning before transplantation in order to induce the expression of HSPs and other DAMPs that are regulatory in their reduced form. Future studies will be needed to establish the best timing and mechanism of therapeutic delivery of regulatory DAMPs following solid organ transplantation to limit AR and CR.

## Author Contributions

HT and GD together generated the text and figures. All authors contributed to the article and approved the submitted version.

## Funding

The authors are supported by NIH grants: R01AR073527(HRT), R01HL122489(HRT), R56AI139327(HRT), T32CA082084(GKD), and F30AI147437(GKD).

## Conflict of Interest

HT is a listed inventor on patent application PCT/US2019/030547 (“MATRIX BOUND VESICLES (MBVS) CONTAINING IL-33 AND THEIR USE”).

The remaining author declares that the research was conducted in the absence of any commercial or financial relationships that could be construed as a potential conflict of interest.

## References

[1] Braza F Brouard S Chadban S Goldstein DR. Role of TLRs and DAMPs in allograft inflammation and transplant outcomes. Nat Rev Nephrol (2016) 12(5):281–90. 10.1038/nrneph.2016.41 PMC7952035 27026348

[2] Todd JL Palmer SM. Danger signals in regulating the immune response to solid organ transplantation. J Clin Invest (2017) 127(7):2464–72. 10.1172/JCI90594 PMC5490743 28530643

[3] Land WG. Endogenous DAMPs, Category I: Constitutively Expressed, Native Molecules (Cat. I DAMPs). Damage-Associated Molecular Patterns in Human Diseases. In: Injury-Induced Innate Immune Responses, vol. 1. Cham: Springer International Publishing (2018). p. 219–68.

[4] Land WG Agostinis P Gasser S Garg AD Linkermann A. Transplantation and Damage-Associated Molecular Patterns (DAMPs). Am J Transplant (2016) 16(12):3338–61. 10.1111/ajt.13963 27421829

[5] Matta BM Reichenbach DK Blazar BR Turnquist HR. Alarmins and Their Receptors as Modulators and Indicators of Alloimmune Responses. Am J Transplant (2017) 17(2):320–7. 10.1111/ajt.13887 PMC5124552 27232285

[6] Gong T Liu L Jiang W Zhou R. DAMP-sensing receptors in sterile inflammation and inflammatory diseases. Nat Rev Immunol (2020) 20(2):95–112. 10.1038/s41577-019-0215-7 31558839

[7] Galluzzi L Vitale I Aaronson SA Abrams JM Adam D Agostinis P. Molecular mechanisms of cell death: recommendations of the Nomenclature Committee on Cell Death 2018. Cell Death Differ (2018) 25(3):486–541. 10.1038/s41418-018-0102-y 29362479 PMC5864239

[8] Huang H Chen HW Evankovich J Yan W Rosborough BR Nace GW. Histones activate the NLRP3 inflammasome in Kupffer cells during sterile inflammatory liver injury. J Immunol (2013) 191(5):2665–79. 10.4049/jimmunol.1202733 PMC3777242 23904166

[9] Xu J Zhang X Monestier M Esmon NL Esmon CT. Extracellular histones are mediators of death through TLR2 and TLR4 in mouse fatal liver injury. J Immunol (2011) 187(5):2626–31. 10.4049/jimmunol.1003930 PMC3159755 21784973

[10] Braza MS van Leent MMT Lameijer M Sanchez-Gaytan BL Arts RJW Perez-Medina C. Inhibiting Inflammation with Myeloid Cell-Specific Nanobiologics Promotes Organ Transplant Acceptance. Immunity (2018) 49(5):819–28.e6. 10.1016/j.immuni.2018.09.008 30413362 PMC6251711

[11] Yang H Hreggvidsdottir HS Palmblad K Wang H Ochani M Li J. A critical cysteine is required for HMGB1 binding to Toll-like receptor 4 and activation of macrophage cytokine release. Proc Natl Acad Sci U S A (2010) 107(26):11942–7. 10.1073/pnas.1003893107 PMC2900689 20547845

[12] Zou H Yang Y Gao M Zhang B Ming B Sun Y. HMGB1 is involved in chronic rejection of cardiac allograft via promoting inflammatory-like mDCs. Am J Transplant (2014) 14(8):1765–77. 10.1111/ajt.12781 24984831

[13] Testro AG Visvanathan K Skinner N Markovska V Crowley P Angus PW. Acute allograft rejection in human liver transplant recipients is associated with signaling through toll-like receptor 4. J Gastroenterol Hepatol (2011) 26(1):155–63. 10.1111/j.1440-1746.2010.06324.x 21175809

[14] Methe H Zimmer E Grimm C Nabauer M Koglin J. Evidence for a role of toll-like receptor 4 in development of chronic allograft rejection after cardiac transplantation. Transplantation (2004) 78(9):1324–31. 10.1097/01.TP.0000137930.40597.03 15548971

[15] Kruger B Krick S Dhillon N Lerner SM Ames S Bromberg JS. Donor Toll-like receptor 4 contributes to ischemia and reperfusion injury following human kidney transplantation. Proc Natl Acad Sci U S A (2009) 106(9):3390–5. 10.1073/pnas.0810169106 PMC2651292 19218437

[16] Dinarello CA. Introduction to the interleukin-1 family of cytokines and receptors: Drivers of innate inflammation and acquired immunity. Immunol Rev (2018) 281(1):5–7. 10.1111/imr.12624 29248001 PMC5750395

[17] Amores-Iniesta J Barbera-Cremades M Martinez CM Pons JA Revilla-Nuin B Martinez-Alarcon L. Extracellular ATP Activates the NLRP3 Inflammasome and Is an EarlyDanger Signal of Skin Allograft Rejection. Cell Rep (2017) 21(12):3414–26. 10.1016/j.celrep.2017.11.079 PMC5746605 29262323

[18] Vergani A Tezza S D’Addio F Fotino C Liu K Niewczas M. Long-term heart transplant survival by targeting the ionotropicpurinergic receptor P2X7. Circulation (2013) 127(4):463–75. 10.1161/CIRCULATIONAHA.112.123653 PMC3569513 23250993

[19] Apostolova P Zeiser R. The role of danger signals and ectonucleotidases in acutegraft-versus-host disease. Hum Immunol (2016) 77(11):1037–47. 10.1016/j.humimm.2016.02.005 26902992

[20] Idzko M Ferrari D Eltzschig HK. Nucleotide signalling during inflammation.Nature (2014) 509(7500):310–7. 10.1038/nature13085 PMC4222675 24828189

[21] Jiang D Liang J Fan J Yu S Chen S Luo Y. Regulation of lung injury and repair by Toll-like receptors andhyaluronan. Nat Med (2005) 11(11):1173–9. 10.1038/nm1315 16244651

[22] Tesar BM Jiang D Liang J Palmer SM Noble PW Goldstein DR. The role of hyaluronan degradation products as innate alloimmune agonists. Am J Transplant (2006) 6(11):2622–35. 10.1111/j.1600-6143.2006.01537.x 17049055

[23] Cui Y Liu K Monzon-Medina ME Padera RF Wang H George G. Therapeutic lymphangiogenesis ameliorates established acute lung allograft rejection. J Clin Invest (2015) 125(11):4255–68. 10.1172/JCI79693 PMC4639995 26485284

[24] Bianchi ME. DAMPs, PAMPs and alarmins: all we need to know about danger. J Leukoc Biol (2007) 81(1):1–5. 10.1189/jlb.0306164 17032697

[25] Krysko DV Agostinis P Krysko O Garg AD Bachert C Lambrecht BN. Emerging role of damage-associated molecular patterns derived from mitochondria in inflammation. Trends Immunol (2011) 32(4):157–64. 10.1016/j.it.2011.01.005 21334975

[26] Zhang Q Raoof M Chen Y Sumi Y Sursal T Junger W. Circulating mitochondrial DAMPs cause inflammatory responses to injury. Nature (2010) 464(7285):104–7. 10.1038/nature08780 PMC2843437 20203610

[27] Bzowska M Nogiec A Bania K Zygmunt M Zarebski M Dobrucki J. Involvement of cell surface 90 kDa heat shock protein (HSP90) in pattern recognition by human monocyte-derived macrophages. J Leukoc Biol (2017) 102(3):763–74. 10.1189/jlb.2MA0117-019R PMC5557637 28550115

[28] Gardai SJ McPhillips KA Frasch SC Janssen WJ Starefeldt A Murphy-Ullrich JE. Cell-surface calreticulin initiates clearance of viable or apoptotic cells through trans-activation of LRP on the phagocyte. Cell (2005) 123(2):321–34. 10.1016/j.cell.2005.08.032 16239148

[29] Terasaki PI Cecka JM Gjertson DW Takemoto S. High survival rates of kidney transplants from spousal and living unrelated donors. N Engl J Med (1995) 333(6):333–6. 10.1056/NEJM199508103330601 7609748

[30] Mikhalski D Wissing KM Ghisdal L Broeders N Touly M Hoang AD. Cold ischemia is a major determinant of acute rejection and renal graft survival in the modern era of immunosuppression. Transplantation (2008) 85(7 Suppl):S3–9. 10.1097/TP.0b013e318169c29e 18401260

[31] Debout A Foucher Y Trebern-Launay K Legendre C Kreis H Mourad G. Each additional hour of cold ischemia time significantly increases the risk of graft failure and mortality following renal transplantation. Kidney Int (2015) 87(2):343–9. 10.1038/ki.2014.304 25229341

[32] Banner NR Thomas HL Curnow E Hussey JC Rogers CA Bonser RS. The importance of cold and warm cardiac ischemia for survival afterheart transplantation. Transplantation (2008) 86(4):542–7. 10.1097/TP.0b013e31818149b9 18724223

[33] Stehlik J Edwards LB Kucheryavaya AY Benden C Christie JD Dobbels F. The Registry of the International Society for Heart and LungTransplantation: Twenty-eighth Adult Heart Transplant Report–2011. J Heart Lung Transplant (2011) 30(10):1078–94. 10.1016/j.healun.2011.08.003 21962016

[34] Ali JM Davies SE Brais RJ Randle LV Klinck JR Allison ME. Analysis of ischemia/reperfusion injury in time-zero biopsiespredicts liver allograft outcomes. Liver Transpl (2015) 21(4):487–99. 10.1002/lt.24072 25545865

[35] Duffy JP Kao K Ko CY Farmer DG McDiarmid SV Hong JC. Long-term patient outcome and quality of life after livertransplantation: analysis of 20-year survivors. Ann Surg (2010) 252(4):652–61. 10.1097/SLA.0b013e3181f5f23a 20881772

[36] Ochando J Ordikhani F Boros P Jordan S. The innate immune response to allotransplants: mechanisms andtherapeutic potentials. Cell Mol Immunol (2019) 16(4):350–6. 10.1038/s41423-019-0216-2 PMC6462017 30804476

[37] Li C Zhang LM Zhang X Huang X Liu Y Li MQ. Short-term Pharmacological Inhibition of MyD88 Homodimerization by aNovel Inhibitor Promotes Robust Allograft Tolerance in Mouse Cardiac and Skin Transplantation. Transplantation (2017) 101(2):284–93. 10.1097/TP.0000000000001471 27607533

[38] Goldstein DR Tesar BM Akira S Lakkis FG. Critical role of the Toll-like receptor signal adaptor protein MyD88in acute allograft rejection. J Clin Invest (2003) 111(10):1571–8. 10.1172/JCI17573 PMC155048 12750407

[39] Wu H Noordmans GA O’Brien MR Ma J Zhao CY Zhang GY. Absence of MyD88 signaling induces donor-specific kidney allografttolerance. J Am Soc Nephrol (2012) 23(10):1701–16. 10.1681/ASN.2012010052 PMC3458459 22878960

[40] Lerret NM Li T Wang JJ Kang HK Wang S Wang X. Recipient Myd88 Deficiency Promotes Spontaneous Resolution of KidneyAllograft Rejection. J Am Soc Nephrol (2015) 26(11):2753–64. 10.1681/ASN.2014080813 PMC4625673 25788530

[41] Zhang X Beduhn M Zheng X Lian D Chen D Li R. Induction of alloimmune tolerance in heart transplantation throughgene silencing of TLR adaptors. Am J Transplant (2012) 12(10):2675–88. 10.1111/j.1600-6143.2012.04196.x 22823145

[42] He WT Zhang LM Li C Li SY Ding ZC Fang ZM. Short-term MyD88 inhibition ameliorates cardiac graft rejection andpromotes donor-specific hyporesponsiveness of skin grafts in mice. Transpl Int (2016) 29(8):941–52. 10.1111/tri.12789 27125343

[43] Barochia A Solomon S Cui X Natanson C Eichacker PQ. Eritoran tetrasodium (E5564) treatment for sepsis: review ofpreclinical and clinical studies. Expert Opin Drug Metab Toxicol (2011) 7(4):479–94. 10.1517/17425255.2011.558190 PMC3065179 21323610

[44] Opal SM Laterre PF Francois B LaRosa SP Angus DC Mira JP. Effect of eritoran, an antagonist of MD2-TLR4, on mortality inpatients with severe sepsis: the ACCESS randomized trial. JAMA (2013) 309(11):1154–62. 10.1001/jama.2013.2194 23512062

[45] Dunn-Siegrist I Leger O Daubeuf B Poitevin Y Depis F Herren S. Pivotal involvement of Fcgamma receptor IIA in the neutralization oflipopolysaccharide signaling via a potent novel anti-TLR4 monoclonal antibody 15C1. J Biol Chem (2007) 282(48):34817–27. 10.1074/jbc.M706440200 17921137

[46] Monnet E Lapeyre G Poelgeest EV Jacqmin P Graaf K Reijers J. Evidence of NI-0101 pharmacological activity, an anti-TLR4 antibody,in a randomized phase I dose escalation study in healthy volunteers receiving LPS. Clin Pharmacol Ther (2017) 101(2):200–8. 10.1002/cpt.522 27706798

[47] Monnet E Choy EH McInnes I Kobakhidze T de Graaf K Jacqmin P. Efficacy and safety of NI-0101, an anti-toll-like receptor 4monoclonal antibody, in patients with rheumatoid arthritis after inadequate response to methotrexate: a phase II study. Ann Rheum Dis (2020) 79(3):316–23. 10.1136/annrheumdis-2019-216487 31892533

[48] Miller RM. (Oct. 2012-June 30, 2016). Placebo-controlled study to evaluate the safety and efficacy of OPN-305 in preventing delayed renal graft function. Identifier NCT01794663. https://www.clinicaltrials.gov/ct2/show/NCT01794663?term1/4opsona&rank1/41.

[49] Farrar CA Keogh B McCormack W O’Shaughnessy A Parker A Reilly M. Inhibition of TLR2 promotes graft function in a murine model ofrenal transplant ischemia-reperfusion injury. FASEB J (2012) 26(2):799–807. 10.1096/fj.11-195396 22042224

[50] Arslan F Houtgraaf JH Keogh B Kazemi K de Jong R McCormack WJ. Treatment with OPN-305, a humanized anti-Toll-Like receptor-2antibody, reduces myocardial ischemia/reperfusion injury in pigs. Circ Cardiovasc Interv (2012) 5(2):279–87. 10.1161/CIRCINTERVENTIONS.111.967596 22354933

[51] Reilly M Miller RM Thomson MH Patris V Ryle P McLoughlin L. Randomized, double-blind, placebo-controlled, dose-escalating phaseI, healthy subjects study of intravenous OPN-305, a humanized anti-TLR2 antibody. Clin Pharmacol Ther (2013) 94(5):593–600. 10.1038/clpt.2013.150 23880971 PMC3805945

[52] Carey B. Irish biotech firm Opsona Therapeutics pops its final pill. The Sunday Times. Times Newspapers Limited, England. England: Times Newspapers Limited (2019). Available at: https://www.thetimes.co.uk/article/irish-biotech-firm-opsona-therapeutics-pops-its-final-pill-jx5nrgj99.

[53] Thomson AW. MyD88 Inhibitors and the Continuing Challenge of TLRAntagonism. Transplantation (2017) 101(2):230–1. 10.1097/TP.0000000000001565 27941436

[54] Lin N Simon MC. Hypoxia-inducible factors: key regulators of myeloid cells during inflammation. J Clin Invest (2016) 126(10):3661–71. 10.1172/JCI84426 PMC5096831 27599290

[55] Cramer T Yamanishi Y Clausen BE Forster I Pawlinski R Mackman N. HIF-1alpha is essential for myeloid cell-mediated inflammation. Cell (2003) 112(5):645–57. 10.1016/S0092-8674(03)00154-5 PMC4480774 12628185

[56] O’Neill LA Pearce EJ. Immunometabolism governs dendritic cell and macrophage function. J Exp Med (2016) 213(1):15–23. 10.1084/jem.20151570 26694970 PMC4710204

[57] Eming SA Wynn TA Martin P. Inflammation and metabolism in tissue repair and regeneration. Science (2017) 356(6342):1026–30. 10.1126/science.aam7928 28596335

[58] Russell DG Huang L VanderVen BC. Immunometabolism at the interface between macrophages and pathogens. Nat Rev Immunol (2019) 19(5):291–304. 10.1038/s41577-019-0124-9 30679807 PMC7032560

[59] Singer BD Chandel NS. Immunometabolism of pro-repair cells. J Clin Invest (2019) 129(7):2597–607. 10.1172/JCI124613 PMC6597201 31081802

[60] Bailey JD Diotallevi M Nicol T McNeill E Shaw A Chuaiphichai S. Nitric Oxide Modulates Metabolic Remodeling in Inflammatory Macrophages through TCA Cycle Regulation and Itaconate Accumulation. Cell Rep (2019) 28(1):218–30.e7. 10.1016/j.celrep.2019.06.018 31269442 PMC6616861

[61] Huang SC Everts B Ivanova Y O’Sullivan D Nascimento M Smith AM. Cell-intrinsic lysosomal lipolysis is essential for alternative activation of macrophages. Nat Immunol (2014) 15(9):846–55. 10.1038/ni.2956 PMC4139419 25086775

[62] Tannahill GM Curtis AM Adamik J Palsson-McDermott EM McGettrick AF Goel G. Succinate is an inflammatory signal that induces IL-1beta through HIF-1alpha. Nature (2013) 496(7444):238–42. 10.1038/nature11986 PMC4031686 23535595

[63] Jantsch J Wiese M Schodel J Castiglione K Glasner J Kolbe S. Toll-like receptor activation and hypoxia use distinct signaling pathways to stabilize hypoxia-inducible factor 1alpha (HIF1A) and result in differential HIF1A-dependent gene expression. J Leukoc Biol (2011) 90(3):551–62. 10.1189/jlb.1210683 21685248

[64] Jiang X Khan MA Tian W Beilke J Natarajan R Kosek J. Adenovirus-mediated HIF-1alpha gene transfer promotes repair of mouse airway allograft microvasculature and attenuates chronic rejection. J Clin Invest (2011) 121(6):2336–49. 10.1172/JCI46192 PMC3104770 21606594

[65] Loor G Schumacker PT. Role of hypoxia-inducible factor in cell survival during myocardial ischemia-reperfusion. Cell Death Differ (2008) 15(4):686–90. 10.1038/cdd.2008.13 18259200

[66] Amaral N Okonko DO. Mitigation of myocardial ischemia-reperfusion injury via HIF-1alpha-frataxin signaling. Am J Physiol Heart Circ Physiol (2015) 309(5):H728–30. 10.1152/ajpheart.00553.2015 PMC4591396 26209059

[67] Xu H Abuduwufuer A Lv W Zhou Z Yang Y Zhang C. The role of HIF-1alpha-VEGF pathway in bronchiolitis obliterans after lung transplantation. J Cardiothorac Surg (2019) 14(1):27. 10.1186/s13019-019-0832-z 30696477 PMC6352448

[68] Ip WKE Hoshi N Shouval DS Snapper S Medzhitov R. Anti-inflammatory effect of IL-10 mediated by metabolic reprogramming of macrophages. Science (2017) 356(6337):513–9. 10.1126/science.aal3535 PMC6260791 28473584

[69] He Y Hara H Nunez G. Mechanism and Regulation of NLRP3 Inflammasome Activation. Trends Biochem Sci (2016) 41(12):1012–21. 10.1016/j.tibs.2016.09.002 PMC5123939 27669650

[70] Chen GY Tang J Zheng P Liu Y. CD24 and Siglec-10 selectively repress tissue damage-induced immune responses. Science (2009) 323(5922):1722–5. 10.1126/science.1168988 PMC2765686 19264983

[71] Macauley MS Crocker PR Paulson JC. Siglec-mediated regulation of immune cell function in disease. Nat Rev Immunol (2014) 14(10):653–66. 10.1038/nri3737 PMC4191907 25234143

[72] Toubai T Hou G Mathewson N Liu C Wang Y Oravecz-Wilson K. Siglec-G-CD24 axis controls the severity of graft-versus-host disease in mice. Blood (2014) 123(22):3512–23. 10.1182/blood-2013-12-545335 PMC4041170 24695850

[73] Zheng P Liu Y Chen H Devenport M Reddy P Farag S. Targeting Danger Associated Molecular Pattern (DAMP) with CD24Fc to Reduce Acute Gvhd: Study Design on a Randomized Double Blind Placebo Controlled Phase III Clinical Trial (CATHY Study). Biol Blood Marrow Transplant (2020) 26(3):S180–S1. 10.1016/j.bbmt.2019.12.741

[74] Arpaia N Green JA Moltedo B Arvey A Hemmers S Yuan S. A Distinct Function of Regulatory T Cells in Tissue Protection. Cell (2015) 162(5):1078–89. 10.1016/j.cell.2015.08.021 PMC4603556 26317471

[75] Burzyn D Kuswanto W Kolodin D Shadrach JL Cerletti M Jang Y. A special population of regulatory T cells potentiates muscle repair. Cell (2013) 155(6):1282–95. 10.1016/j.cell.2013.10.054 PMC3894749 24315098

[76] Kuswanto W Burzyn D Panduro M Wang KK Jang YC Wagers AJ. Poor Repair of Skeletal Muscle in Aging Mice Reflects a Defect in Local, Interleukin-33-Dependent Accumulation of Regulatory T Cells. Immunity (2016) 44(2):355–67. 10.1016/j.immuni.2016.01.009 PMC4764071 26872699

[77] Liu Q Dwyer GK Zhao Y Li H Mathews LR Chakka AB. IL-33-mediated IL-13 secretion by ST2+ Tregs controls inflammation after lung injury. JCI Insight (2019) 4(6). 10.1172/jci.insight.123919 PMC6482994 30779711

[78] Li T Zhang Z Bartolacci JG Dwyer GK Liu Q Mathews LR. Graft IL-33 regulates infiltrating macrophages to protect against chronic rejection. J Clin Invest (2020). 10.1172/JCI133008 PMC7524467 32644975

[79] Brunner SM Schiechl G Falk W Schlitt HJ Geissler EK Fichtner-Feigl S. Interleukin-33 prolongs allograft survival during chronic cardiac rejection. Transpl Int (2011) 24(10):1027–39. 10.1111/j.1432-2277.2011.01306.x 21797940

[80] Gajardo T Morales RA Campos-Mora M Campos-Acuna J Pino-Lagos K. Exogenous interleukin-33 targets myeloid-derived suppressor cells and generates periphery-induced Foxp3(+) regulatory T cells in skin-transplanted mice. Immunology (2015) 146(1):81–8. 10.1111/imm.12483 PMC4552503 25988395

[81] Kawai K Uchiyama M Hester J Issa F. IL-33 drives the production of mouse regulatory T cells with enhanced in vivo suppressive activity in skin transplantation. Am J Transplant (2020). 10.1111/ajt.16266 PMC7613121 33314772

[82] Matta BM Reichenbach DK Zhang X Mathews L Koehn BH Dwyer GK. Peri-alloHCT IL-33 administration expands recipient T-regulatory cells that protect mice against acute GVHD. Blood (2016) 128(3):427–39. 10.1182/blood-2015-12-684142 PMC4957164 27222477

[83] Jones Q Voegeli TS Li G Chen Y Currie RW. Heat shock proteins protect against ischemia and inflammation through multiple mechanisms. Inflammation Allergy Drug Targets (2011) 10(4):247–59. 10.2174/187152811796117726 21539516

[84] Seemampillai B Germack R Felkin LE McCormack A Rose ML. Heat shock protein-27 delays acute rejection after cardiac transplantation: an experimental model. Transplantation (2014) 98(1):29–38. 10.1097/TP.0000000000000170 24879379 PMC4164282

[85] Elias D Meilin A Ablamunits V Birk OS Carmi P Konen-Waisman S. Hsp60 peptide therapy of NOD mouse diabetes induces a Th2 cytokine burst and downregulates autoimmunity to various beta-cell antigens. Diabetes (1997) 46(5):758–64. 10.2337/diabetes.46.5.758 9133541

[86] Luna E Postol E Caldas C Benvenuti LA Rodrigues JMJ Lima K. Treatment with encapsulated Hsp60 peptide (p277) prolongs skin graft survival in a murine model of minor antigen disparity. Scand J Immunol (2007) 66(1):62–70. 10.1111/j.1365-3083.2007.01951.x 17587347

[87] Borges TJ Porto BN Teixeira CA Rodrigues M Machado FD Ornaghi AP. Prolonged survival of allografts induced by mycobacterial Hsp70 is dependent on CD4+CD25+ regulatory T cells. PloS One (2010) 5(12):e14264. 10.1371/journal.pone.0014264 21170379 PMC2999527

[88] Lopes RL Borges TJ Araujo JF Pinho NG Bergamin LS Battastini AM. Extracellular mycobacterial DnaK polarizes macrophages to the M2-like phenotype. PloS One (2014) 9(11):e113441. 10.1371/journal.pone.0113441 25419575 PMC4242626

[89] Borges TJ Lang BJ Lopes RL Bonorino C. Modulation of Alloimmunity by Heat Shock Proteins. Front Immunol (2016) 7:303. 10.3389/fimmu.2016.00303 27555846 PMC4977877

[90] Dick SA Macklin JA Nejat S Momen A Clemente-Casares X Althagafi MG. Self-renewing resident cardiac macrophages limit adverse remodeling following myocardial infarction. Nat Immunol (2019) 20(1):29–39. 10.1038/s41590-018-0272-2 30538339 PMC6565365

[91] Lim HY Lim SY Tan CK Thiam CH Goh CC Carbajo D. Hyaluronan Receptor LYVE-1-Expressing Macrophages Maintain Arterial Tone through Hyaluronan-Mediated Regulation of Smooth Muscle Cell Collagen. Immunity (2018) 49(2):326–41.e7. 10.1016/j.immuni.2018.12.009 30054204

[92] Bajpai G Bredemeyer A Li W Zaitsev K Koenig AL Lokshina I. Tissue Resident CCR2- and CCR2+ Cardiac Macrophages Differentially Orchestrate Monocyte Recruitment and Fate Specification Following Myocardial Injury. Circ Res (2019) 124(2):263–78. 10.1161/CIRCRESAHA.118.314028 PMC6626616 30582448

[93] Jiang D Liang J Noble PW. Hyaluronan as an immune regulator in human diseases. Physiol Rev (2011) 91(1):221–64. 10.1152/physrev.00052.2009 PMC3051404 21248167

[94] Petz A Grandoch M Gorski DJ Abrams M Piroth M Schneckmann R. Cardiac Hyaluronan Synthesis Is Critically Involved in the Cardiac Macrophage Response and Promotes Healing After Ischemia Reperfusion Injury. Circ Res (2019) 124(10):1433–47. 10.1161/CIRCRESAHA.118.313285 30916618

[95] McArthur S Juban G Gobbetti T Desgeorges T Theret M Gondin J. Annexin A1 drives macrophage skewing to accelerate muscle regeneration through AMPK activation. J Clin Invest (2020) 130(3):1156–67. 10.1172/JCI124635 PMC7269594 32015229

[96] Teixeira RA Mimura KK Araujo LP Greco KV Oliani SM. The essential role of annexin A1 mimetic peptide in the skin allograft survival. J Tissue Eng Regener Med (2016) 10(2):E44–53. 10.1002/term.1773 23897745

[97] La M D’Amico M Bandiera S Di Filippo C Oliani SM Gavins FN. Annexin 1 peptides protect against experimental myocardial ischemia-reperfusion: analysis of their mechanism of action. FASEB J (2001) 15(12):2247–56. 10.1096/fj.01-0196com 11641252

[98] Qin C Buxton KD Pepe S Cao AH Venardos K Love JE. Reperfusion-induced myocardial dysfunction is prevented by endogenous annexin-A1 and its N-terminal-derived peptide Ac-ANX-A1(2-26). Br J Pharmacol (2013) 168(1):238–52. 10.1111/j.1476-5381.2012.02176.x PMC3570018 22924634

[99] Qin CX Finlayson SB Al-Sharea A Tate M De Blasio MJ Deo M. Endogenous Annexin-A1 Regulates Haematopoietic Stem Cell Mobilisation and Inflammatory Response Post Myocardial Infarction in Mice In Vivo. Sci Rep (2017) 7(1):16615. 10.1038/s41598-017-16317-1 29192208 PMC5709412

[100] Serhan CN. Pro-resolving lipid mediators are leads for resolution physiology. Nature (2014) 510(7503):92–101. 10.1038/nature13479 24899309 PMC4263681

[101] Buckley CD Gilroy DW Serhan CN. Proresolving lipid mediators and mechanisms in the resolution of acute inflammation. Immunity (2014) 40(3):315–27. 10.1016/j.immuni.2014.02.009 PMC4004957 24656045

[102] Ramon S Bancos S Serhan CN Phipps RP. Lipoxin A(4) modulates adaptive immunity by decreasing memory B-cell responses via an ALX/FPR2-dependent mechanism. Eur J Immunol (2014) 44(2):357–69. 10.1002/eji.201343316 PMC3966998 24166736

[103] Levy BD Zhang QY Bonnans C Primo V Reilly JJ Perkins DL. The endogenous pro-resolving mediators lipoxin A4 and resolvin E1 preserve organ function in allograft rejection. Prostaglandins Leukot Essent Fatty Acids (2011) 84(1-2):43–50. 10.1016/j.plefa.2010.09.002 20869861 PMC3019284

[104] Dalli J Zhu M Vlasenko NA Deng B Haeggstrom JZ Petasis NA. The novel 13S,14S-epoxy-maresin is converted by human macrophages to maresin 1 (MaR1), inhibits leukotriene A4 hydrolase (LTA4H), and shifts macrophage phenotype. FASEB J (2013) 27(7):2573–83. 10.1096/fj.13-227728 PMC3688739 23504711

[105] Navarro-Xavier RA Newson J Silveira VL Farrow SN Gilroy DW Bystrom J. A new strategy for the identification of novel molecules with targeted proresolution of inflammation properties. J Immunol (2010) 184(3):1516–25. 10.4049/jimmunol.0902866 20032295

[106] Qiu Y Wu Y Zhao H Sun H Gao S. Maresin 1 mitigates renal ischemia/reperfusion injury in mice via inhibition of the TLR4/MAPK/NF-kappaB pathways and activation of the Nrf2 pathway. Drug Des Devel Ther (2019) 13:739–45. 10.2147/DDDT.S188654 PMC6388965 30863013

[107] Soto G Rodriguez MJ Fuentealba R Treuer AV Castillo I Gonzalez DR. Maresin 1, a Proresolving Lipid Mediator, Ameliorates Liver Ischemia-Reperfusion Injury and Stimulates Hepatocyte Proliferation in Sprague-Dawley Rats. Int J Mol Sci (2020) 21(2). 10.3390/ijms21020540 PMC7014175 31952110

[108] Sun Q Wu Y Zhao F Wang J. Maresin 1 Ameliorates Lung Ischemia/Reperfusion Injury by Suppressing Oxidative Stress via Activation of the Nrf-2-Mediated HO-1 Signaling Pathway. Oxid Med Cell Longev (2017) 2017:9634803. 10.1155/2017/9634803 28751936 PMC5511669

[109] Chiurchiu V Leuti A Dalli J Jacobsson A Battistini L Maccarrone M. Proresolving lipid mediators resolvin D1, resolvin D2, and maresin 1are critical in modulating T cell responses. Sci Transl Med (2016) 8(353):353ra111. 10.1126/scitranslmed.aaf7483 PMC5149396 27559094

[110] Toubai T Rossi C Oravecz-Wilson K Zajac C Liu C Braun T. Siglec-G represses DAMP-mediated effects on T cells.JCI Insight (2017) 2(14). 10.1172/jci.insight.92293 PMC5518560 28724800

[111] Gautier EL Shay T Miller J Greter M Jakubzick C Ivanov S. Gene-expression profiles and transcriptional regulatory pathwaysthat underlie the identity and diversity of mouse tissue macrophages. Nat Immunol (2012) 13(11):1118–28. 10.1038/ni.2419 PMC3558276 23023392

[112] Bajpai G Schneider C Wong N Bredemeyer A Hulsmans M Nahrendorf M. The human heart contains distinct macrophage subsets with divergentorigins and functions. Nat Med (2018) 24(8):1234–45. 10.1038/s41591-018-0059-x PMC6082687 29892064

[113] Meziani L Deutsch E Mondini M. Macrophages in radiation injury: a new therapeutictarget. Oncoimmunology (2018) 7(10):e1494488. 10.1080/2162402X.2018.1494488 30288363 PMC6169587

[114] Lee S Huen S Nishio H Nishio S Lee HK Choi BS. Distinct macrophage phenotypes contribute to kidney injury and repair. J Am Soc Nephrol (2011) 22(2):317–26. 10.1681/ASN.2009060615 PMC3029904 21289217

[115] Heidt T Courties G Dutta P Sager HB Sebas M Iwamoto Y. Differential contribution of monocytes to heart macrophages in steady-state and after myocardial infarction. Circ Res (2014) 115(2):284–95. 10.1161/CIRCRESAHA.115.303567 PMC4082439 24786973

[116] Wynn TA Vannella KM. Macrophages in Tissue Repair, Regeneration, and Fibrosis. Immunity (2016) 44(3):450–62. 10.1016/j.immuni.2016.02.015 PMC4794754 26982353

[117] Aurora AB Porrello ER Tan W Mahmoud AI Hill JA Bassel-Duby R. Macrophages are required for neonatal heart regeneration. J Clin Invest (2014) 124(3):1382–92. 10.1172/JCI72181 PMC3938260 24569380

[118] Bosurgi L Cao YG Cabeza-Cabrerizo M Tucci A Hughes LD Kong Y. Macrophage function in tissue repair and remodeling requires IL-4 or IL-13 with apoptotic cells. Science (2017) 356(6342):1072–6. 10.1126/science.aai8132 PMC5556699 28495875

[119] Herber DL Cao W Nefedova Y Novitskiy SV Nagaraj S Tyurin VA. Lipid accumulation and dendritic cell dysfunction in cancer. Nat Med (2010) 16(8):880–6. 10.1038/nm.2172 PMC2917488 20622859

[120] Mills EL O’Neill LA. Reprogramming mitochondrial metabolism in macrophages as an anti-inflammatory signal. Eur J Immunol (2016) 46(1):13–21. 10.1002/eji.201445427 26643360

[121] Shiraishi M Shintani Y Shintani Y Ishida H Saba R Yamaguchi A. Alternatively activated macrophages determine repair of the infarcted adult murine heart. J Clin Invest (2016) 126(6):2151–66. 10.1172/JCI85782 PMC4887176 27140396

[122] Kang K Reilly SM Karabacak V Gangl MR Fitzgerald K Hatano B. Adipocyte-derived Th2 cytokines and myeloid PPARdelta regulate macrophage polarization and insulin sensitivity. Cell Metab (2008) 7(6):485–95. 10.1016/j.cmet.2008.04.002 PMC2586840 18522830

[123] Vats D Mukundan L Odegaard JI Zhang L Smith KL Morel CR. Oxidative metabolism and PGC-1beta attenuate macrophage-mediated inflammation. Cell Metab (2006) 4(1):13–24. 10.1016/j.cmet.2006.05.011 16814729 PMC1904486

[124] Rath M Müller I Kropf P Closs EI Munder M. Metabolism via Arginase or Nitric Oxide Synthase: Two Competing Arginine Pathways in Macrophages. Front Immunol (2014) 5(532). 10.3389/fimmu.2014.00532 PMC4209874 25386178

[125] Bronte V Serafini P De Santo C Marigo I Tosello V Mazzoni A. IL-4-induced arginase 1 suppresses alloreactive T cells in tumor-bearing mice. J Immunol (2003) 170(1):270–8. 10.4049/jimmunol.170.1.270 12496409

[126] Highfill SL Rodriguez PC Zhou Q Goetz CA Koehn BH Veenstra R. Bone marrow myeloid-derived suppressor cells (MDSCs) inhibit graft-versus-host disease (GVHD) via an arginase-1-dependent mechanism that is up-regulated by interleukin-13. Blood (2010) 116(25):5738–47. 10.1182/blood-2010-06-287839 PMC3031417 20807889

[127] Broichhausen C Riquelme P Geissler EK Hutchinson JA. Regulatory macrophages as therapeutic targets and therapeutic agents in solid organ transplantation. Curr Opin Organ Transplant (2012) 17(4):332–42. 10.1097/MOT.0b013e328355a979 22790067

[128] Chakarov S Lim HY Tan L Lim SY See P Lum J. Two distinct interstitial macrophage populations coexist across tissues in specific subtissular niches. Science (2019) 363(6432). 10.1126/science.aau0964 30872492

[129] Wildin RS Ramsdell F Peake J Faravelli F Casanova JL Buist N. X-linked neonatal diabetes mellitus, enteropathy and endocrinopathy syndrome is the human equivalent of mouse scurfy. Nat Genet (2001) 27(1):18–20. 10.1038/83707 11137992

[130] Sakaguchi S Yamaguchi T Nomura T Ono M. Regulatory T cells and immune tolerance. Cell (2008) 133(5):775–87. 10.1016/j.cell.2008.05.009 18510923

[131] Romano M Fanelli G Albany CJ Giganti G Lombardi G. Past, Present, and Future of Regulatory T Cell Therapy in Transplantation and Autoimmunity. Front Immunol (2019) 10:43. 10.3389/fimmu.2019.00043 30804926 PMC6371029

[132] Zaiss DMW Gause WC Osborne LC Artis D. Emerging functions of amphiregulin in orchestrating immunity, inflammation, and tissue repair. Immunity (2015) 42(2):216–26. 10.1016/j.immuni.2015.01.020 PMC4792035 25692699

[133] Dial CF Tune MK Doerschuk CM Mock JR. Foxp3(+) Regulatory T Cell Expression of Keratinocyte Growth Factor Enhances Lung Epithelial Proliferation. Am J Respir Cell Mol Biol (2017) 57(2):162–73. 10.1165/rcmb.2017-0019OC PMC5576587 28296468

[134] Liu H Liu L Liu K Bizargity P Hancock WW Visner GA. Reduced cytotoxic function of effector CD8+ T cells is responsible for indoleamine 2,3-dioxygenase-dependent immune suppression. J Immunol (2009) 183(2):1022–31. 10.4049/jimmunol.0900408 19564344

[135] Tiemessen MM Jagger AL Evans HG van Herwijnen MJ John S Taams LS. CD4+CD25+Foxp3+ regulatory T cells induce alternative activation of human monocytes/macrophages. Proc Natl Acad Sci U S A (2007) 104(49):19446–51. 10.1073/pnas.0706832104 PMC2148309 18042719

[136] Liesz A Kleinschnitz C. Regulatory T Cells in Post-stroke Immune Homeostasis. Transl Stroke Res (2016) 7(4):313–21. 10.1007/s12975-016-0465-7 27030356

[137] Weirather J Hofmann UD Beyersdorf N Ramos GC Vogel B Frey A. Foxp3+ CD4+ T cells improve healing after myocardial infarction by modulating monocyte/macrophage differentiation. Circ Res (2014) 115(1):55–67. 10.1161/CIRCRESAHA.115.303895 24786398

[138] Julier Z Park AJ Briquez PS Martino MM. Promoting tissue regeneration by modulating the immune system. Acta Biomater (2017) 53:13–28. 10.1016/j.actbio.2017.01.056 28119112

[139] Serhan CN Levy BD. Resolvins in inflammation: emergence of the pro-resolving superfamily of mediators. J Clin Invest (2018) 128(7):2657–69. 10.1172/JCI97943 PMC6025982 29757195

[140] Basil MC Levy BD. Specialized pro-resolving mediators: endogenous regulators of infection and inflammation. Nat Rev Immunol (2016) 16(1):51–67. 10.1038/nri.2015.4 26688348 PMC5242505

[141] Chandrasekharan JA Sharma-Walia N. Lipoxins: nature’s way to resolve inflammation. J Inflammation Res (2015) 8:181–92. 10.2147/JIR.S90380 PMC4598198 26457057

[142] El Kebir D Jozsef L Pan W Wang L Petasis NA Serhan CN. 15-epi-lipoxin A4 inhibits myeloperoxidase signaling and enhances resolution of acute lung injury. Am J Respir Crit Care Med (2009) 180(4):311–9. 10.1164/rccm.200810-1601OC PMC2731808 19483113

[143] Titos E Rius B Lopez-Vicario C Alcaraz-Quiles J Garcia-Alonso V Lopategi A. Signaling and Immunoresolving Actions of Resolvin D1 in Inflamed Human Visceral Adipose Tissue. J Immunol (2016) 197(8):3360–70. 10.4049/jimmunol.1502522 PMC5101161 27647830

[144] Gavins FN Hickey MJ. Annexin A1 and the regulation of innate and adaptive immunity. Front Immunol (2012) 3:354. 10.3389/fimmu.2012.00354 23230437 PMC3515881

[145] Bode K Bujupi F Link C Hein T Zimmermann S Peiris D. Dectin-1 Binding to Annexins on Apoptotic Cells Induces Peripheral Immune Tolerance via NADPH Oxidase-2. Cell Rep (2019) 29(13):4435–46.e9. 10.1016/j.celrep.2019.11.086 31875551

[146] Riise GC Schersten H Nilsson F Ryd W Andersson BA. Activation of eosinophils and fibroblasts assessed by eosinophil cationic protein and hyaluronan in BAL. Association with acute rejection in lung transplant recipients. Chest (1996) 110(1):89–96. 10.1378/chest.110.1.89 8681673

[147] Todd JL Wang X Sugimoto S Kennedy VE Zhang HL Pavlisko EN. Hyaluronan contributes to bronchiolitis obliterans syndrome and stimulates lung allograft rejection through activation of innate immunity. Am J Respir Crit Care Med (2014) 189(5):556–66. 10.1164/rccm.201308-1481OC PMC3977710 24471427

[148] Johnsson C Tufveson G. Serum hyaluronan–a potential marker of cardiac allograft rejection? J Heart Lung Transplant (2006) 25(5):544–9. 10.1016/j.healun.2005.06.029 16678033

[149] Knoflach A Magee C Denton MD Kim KS Buelow R Hancock WW. Immunomodulatory functions of hyaluronate in the LEW-to-F344 model of chronic cardiac allograft rejection. Transplantation (1999) 67(6):909–14. 10.1097/00007890-199903270-00020 10199742

[150] Zhang W Gao L Qi S Liu D Xu D Peng J. Blocking of CD44-hyaluronic acid interaction prolongs rat allograft survival. Transplantation (2000) 69(4):665–7. 10.1097/00007890-200002270-00032 10708127

[151] Chanmee T Ontong P Itano N. Hyaluronan: A modulator of the tumor microenvironment. Cancer Lett (2016) 375(1):20–30. 10.1016/j.canlet.2016.02.031 26921785

[152] Skandalis SS Karalis TT Chatzopoulos A Karamanos NK. Hyaluronan-CD44 axis orchestrates cancer stem cell functions. Cell Signal (2019) 63:109377. 10.1016/j.cellsig.2019.109377 31362044

[153] Wang F Chen J Shao W Xie B Wang Y Lan T. Anti-CD44 monoclonal antibody inhibits heart transplant rejection mediated by alloantigen-primed CD4(+) memory T cells in nude mice. Immunol Invest (2010) 39(8):807–19. 10.3109/08820139.2010.497833 20718664

[154] Liew FY Girard JP Turnquist HR. Interleukin-33 in health and disease. NatRev Immunol (2016) 16(11):676–89. 10.1038/nri.2016.95 27640624

[155] Roussel L Erard M Cayrol C Girard JP. Molecular mimicry between IL-33 and KSHV for attachment to chromatinthrough the H2A-H2B acidic pocket. EMBO Rep (2008) 9(10):1006–12. 10.1038/embor.2008.145 PMC2572127 18688256

[156] Travers J Rochman M Miracle CE Habel JE Brusilovsky M Caldwell JM. Chromatin regulates IL-33 release and extracellular cytokineactivity. Nat Commun (2018) 9(1):3244. 10.1038/s41467-018-05485-x 30108214 PMC6092330

[157] Hussey GS Dziki JL Lee YC Bartolacci JG Behun M Turnquist HR. Matrix bound nanovesicle-associated IL-33 activates a pro-remodelingmacrophage phenotype via a non-canonical, ST2-independent pathway. J Immunol Regener Med (2019) 3:26–35. 10.1016/j.regen.2019.01.001 PMC6814021 31656879

[158] Turnquist HR Zhao Z Rosborough BR Liu Q Castellaneta A Isse K. IL-33 expands suppressive CD11b+ Gr-1(int) and regulatory T cells,including ST2L+ Foxp3+ cells, and mediates regulatory T cell-dependent promotion of cardiac allograft survival. J Immunol (2011) 187(9):4598–610. 10.4049/jimmunol.1100519 PMC3197898 21949025

[159] Bonilla WV Frohlich A Senn K Kallert S Fernandez M Johnson S. The alarmin interleukin-33 drives protective antiviral CD8(+) T cell responses. Science (2012) 335(6071):984–9. 10.1126/science.1215418 22323740

[160] Reichenbach DK Schwarze V Matta BM Tkachev V Lieberknecht E Liu Q. The IL-33/ST2 axis augments effector T-cell responses during acute GVHD. Blood (2015) 125(20):3183–92. 10.1182/blood-2014-10-606830 PMC4432012 25814531

[161] Baumann C Bonilla WV Frohlich A Helmstetter C Peine M Hegazy AN. T-bet- and STAT4-dependent IL-33 receptor expression directly promotes antiviral Th1 cell responses. Proc Natl Acad Sci U S A (2015) 112(13):4056–61. 10.1073/pnas.1418549112 PMC4386370 25829541

[162] Matta BM Lott JM Mathews LR Liu Q Rosborough BR Blazar BR. IL-33 is an unconventional Alarmin that stimulates IL-2 secretion by dendritic cells to selectively expand IL-33R/ST2+ regulatory T cells. J Immunol (2014) 193(8):4010–20. 10.4049/jimmunol.1400481 PMC4185240 25217167

[163] Stier MT Mitra R Nyhoff LE Goleniewska K Zhang J Puccetti MV. IL-33 Is a Cell-Intrinsic Regulator of Fitness during Early B Cell Development. J Immunol (2019) 203(6):1457–67. 10.4049/jimmunol.1900408 PMC6736727 31391233

[164] Dahlgren MW Jones SW Cautivo KM Dubinin A Ortiz-Carpena JF Farhat S. Adventitial Stromal Cells Define Group 2 Innate Lymphoid Cell Tissue Niches. Immunity (2019) 50(3):707–22.e6. 10.1016/j.immuni.2019.02.002 30824323 PMC6553479

[165] Turnquist HR Cardinal J Macedo C Rosborough BR Sumpter TL Geller DA. mTOR and GSK-3 shape the CD4+ T-cell stimulatory and differentiation capacity of myeloid DCs after exposure to LPS. Blood (2010) 115(23):4758–69. 10.1182/blood-2009-10-251488 PMC2890188 20335217

[166] Panduro M Benoist C Mathis D. Tissue Tregs. Annu Rev Immunol (2016) 34:609–33. 10.1146/annurev-immunol-032712-095948 PMC4942112 27168246

[167] Lechner AJ Driver IH Lee J Conroy CM Nagle A Locksley RM. Recruited Monocytes and Type 2 Immunity Promote Lung Regeneration following Pneumonectomy. Cell Stem Cell (2017) 21(1):120–34.e7. 10.1016/j.stem.2017.03.024 28506464 PMC5501755

[168] Kolodin D van Panhuys N Li C Magnuson AM Cipolletta D Miller CM. Antigen- and cytokine-driven accumulation of regulatory T cells in visceral adipose tissue of lean mice. Cell Metab (2015) 21(4):543–57. 10.1016/j.cmet.2015.03.005 PMC4747251 25863247

[169] Miller AM Asquith DL Hueber AJ Anderson LA Holmes WM McKenzie AN. Interleukin-33 induces protective effects in adipose tissueinflammation during obesity in mice. Circ Res (2010) 107(5):650–8. 10.1161/CIRCRESAHA.110.218867 PMC4254700 20634488

[170] Molofsky AB Nussbaum JC Liang HE Van Dyken SJ Cheng LE Mohapatra A. Innate lymphoid type 2 cells sustain visceral adipose tissueeosinophils and alternatively activated macrophages. J Exp Med (2013) 210(3):535–49. 10.1084/jem.20121964 PMC3600903 23420878

[171] Zhuang Q Liu Q Divito SJ Zeng Q Yatim KM Hughes AD. Graft-infiltrating host dendritic cells play a key role in organtransplant rejection. Nat Commun (2016) 7:12623. 10.1038/ncomms12623 27554168 PMC4999515

[172] Dai H Friday AJ Abou-Daya KI Williams AL Mortin-Toth S Nicotra ML. Donor SIRPalpha polymorphism modulates the innate immune response toallogeneic grafts. Sci Immunol (2017) 2(12). 10.1126/sciimmunol.aam6202 PMC5653256 28783664

[173] Oberbarnscheidt MH Zeng Q Li Q Dai H Williams AL Shlomchik WD. Non-self recognition by monocytes initiates allograftrejection. J Clin Invest (2014) 124(8):3579–89. 10.1172/JCI74370 PMC4109551 24983319

[174] Dai H Lan P Zhao D Abou-Daya K Liu W Chen W. PIRs mediate innate myeloid cell memory to nonself MHC molecules. Science (2020) 368(6495):1122–7. 10.1126/science.aax4040 PMC7379379 32381589

[175] Lever JM Hull TD Boddu R Pepin ME Black LM Adedoyin OO. Resident macrophages reprogram toward a developmental state after acute kidney injury. JCI Insight (2019) 4(2). 10.1172/jci.insight.125503 PMC6413788 30674729

[176] Fletcher AL Baker AT Lukacs-Kornek V Knoblich K. The fibroblastic T cell niche in lymphoid tissues. Curr Opin Immunol (2020) 64:110–6. 10.1016/j.coi.2020.04.007 32497868

[177] Gajewski TF Schreiber H Fu YX. Innate and adaptive immune cells in the tumor microenvironment. Nat Immunol (2013) 14(10):1014–22. 10.1038/ni.2703 PMC4118725 24048123

[178] Mendez-Ferrer S Bonnet D Steensma DP Hasserjian RP Ghobrial IM Gribben JG. Bone marrow niches in haematological malignancies. Nat Rev Cancer (2020) 20(5):285–98. 10.1038/s41568-020-0245-2 PMC9912977 32112045

[179] Jardine L Cytlak U Gunawan M Reynolds G Green K Wang XN. Donor monocyte-derived macrophages promote human acute graft-versus-host disease. J Clin Invest (2020) 130(9):4574–86. 10.1172/JCI133909 PMC7456218 32453711

[180] Halloran PF Reeve J Aliabadi AZ Cadeiras M Crespo-Leiro MG Deng M. Exploring the cardiac response to injury in heart transplant biopsies. JCI Insight (2018) 3(20). 10.1172/jci.insight.123674 PMC6237487 30333303

[181] Mahlakoiv T Flamar AL Johnston LK Moriyama S Putzel GG Bryce PJ. Stromal cells maintain immune cell homeostasis in adipose tissue via production of interleukin-33. Sci Immunol (2019) 4(35). 10.1126/sciimmunol.aax0416 PMC6766755 31053655

[182] Cohen ES Scott IC Majithiya JB Rapley L Kemp BP England E. Oxidation of the alarmin IL-33 regulates ST2-dependent inflammation. Nat Commun (2015) 6:8327. 10.1183/13993003.congress-2015.OA292 26365875 PMC4579851

[183] Yang H Wang H Ju Z Ragab AA Lundback P Long W. MD-2 is required for disulfide HMGB1-dependent TLR4 signaling. J Exp Med (2015) 212(1):5–14. 10.1084/jem.20141318 25559892 PMC4291531

[184] Tirone M Tran NL Ceriotti C Gorzanelli A Canepari M Bottinelli R. High mobility group box 1 orchestrates tissue regeneration via CXCR4. J Exp Med (2018) 215(1):303–18. 10.1084/jem.20160217 PMC5748844 29203538

[185] Venereau E Casalgrandi M Schiraldi M Antoine DJ Cattaneo A De Marchis F. Mutually exclusive redox forms of HMGB1 promote cell recruitment or proinflammatory cytokine release. J Exp Med (2012) 209(9):1519–28. 10.1084/jem.20120189 PMC3428943 22869893

[186] Huber R Meier B Otsuka A Fenini G Satoh T Gehrke S. Tumour hypoxia promotes melanoma growth and metastasis via High Mobility Group Box-1 and M2-like macrophages. Sci Rep (2016) 6:29914. 10.1038/srep29914 27426915 PMC4947927

[187] Xu J Guardado J Hoffman R Xu H Namas R Vodovotz Y. IL33-mediated ILC2 activation and neutrophil IL5 production in the lung response after severe trauma: A reverse translation study from a human cohort to a mouse trauma model. PloS Med (2017) 14(7):e1002365. 10.1371/journal.pmed.1002365 28742815 PMC5526517

[188] Ardehali A Esmailian F Deng M Soltesz E Hsich E Naka Y. Ex-vivo perfusion of donor hearts for human heart transplantation (PROCEED II): a prospective, open-label, multicentre, randomised non-inferiority trial. Lancet (2015) 385(9987):2577–84. 10.1016/S0140-6736(15)60261-6 25888086

[189] Bishawi M Roan JN Milano CA Daneshmand MA Schroder JN Chiang Y. A normothermic ex vivo organ perfusion delivery method for cardiac transplantation gene therapy. Sci Rep (2019) 9(1):8029. 10.1038/s41598-019-43737-y 31142753 PMC6541710

